# Establishment and application of an RNAi system in *Pichia pastoris*

**DOI:** 10.3389/fbioe.2025.1548187

**Published:** 2025-03-05

**Authors:** Shupeng Ruan, Chenfeng He, Aoxue Wang, Ying Lin, Shuli Liang

**Affiliations:** ^1^ Guangdong Key Laboratory of Fermentation and Enzyme Engineering, School of Biology and Biological Engineering, South China University of Technology, Guangzhou, China; ^2^ Guangdong Research Center of Industrial Enzyme and Green Manufacturing Technology, School of Biology and Biological Engineering, South China University of Technology, Guangzhou, China

**Keywords:** *P. pastoris*, RNA interference, EGFP, gene regulation, metabolic engineering, protein secretion

## Abstract

**Introduction:**

Reducing endogenous gene expression is key in microbial metabolic engineering. Traditional methods for gene knockout or suppression can be slow and complex. RNA interference (RNAi) provides a faster way to regulate gene expression using plasmids with hairpin RNA. This study examines single- and double-gene suppression in *P. pastoris*, a common system for expressing heterologous proteins. We also use reporter strains displaying EGFP on the cell surface to identify factors affecting protein secretion.

**Methods:**

We established an RNAi system in *P. pastoris* by introducing plasmids containing hairpin RNA targeting specific genes. Reporter strains expressing *EGFP* on the cell surface were used to monitor the impact of gene suppression on protein secretion. Genes such as *YAP1*, *YPS1*, *PRB1*, and *PEP4* were targeted for RNAi. Additionally, RNAi was applied to inhibit fatty acid synthesis to improve the conversion of malonyl-CoA to 3-hydroxypropionate (3-HP).

**Results:**

Suppressing *YAP1* and *YPS1* reduced *EGFP* display by 83% and 48.8%, respectively. In contrast, suppressing *PRB1* and *PEP4* increased *EGFP* display by 33.8% and 26.5%, respectively. These findings show that regulating endogenous genes can significantly impact protein secretion in *P. pastoris*. Furthermore, RNAi inhibition of fatty acid synthesis improved 3-HP production.

**Discussion:**

This study demonstrates the successful establishment of an RNAi system in *P. pastoris*, enabling efficient gene suppression for metabolic engineering. RNAi offers a faster and more efficient method for regulating gene expression, improving heterologous protein secretion and 3-HP production. This system is a valuable tool for optimizing *P. pastoris* as a microbial cell factory, with strong potential for industrial applications.

## 1 Introduction

*Pichia pastoris* is a methylotrophic yeast widely used for the production of recombinant proteins due to its robust promoters, post-translational modification capabilities, ease of culture, and potential for high-density fermentation (Ahmad et al., 2014; Liu et al., 2022; Zha et al., 2023). Recently, *P. pastoris* has become a leading industrial platform for producing recombinant proteins, especially in the biopharmaceutical and industrial enzyme sectors. As a “generally regarded as safe” (GRAS) microorganism, *P. pastoris* has proven to be a valuable tool in biotechnology. According to data from the RCT platform (www.pichia.com), over 5000 distinct proteins have been successfully expressed in this yeast (Schwarzhans et al., 2017).

Typical strategies to enhance exogenous protein expression in *P. pastoris* include promoter engineering (Nong et al., 2020; Lai et al., 2024; Zhou et al., 2023), signal peptide modification (Li et al., 2015), increasing copy number (Liu et al., 2020; Wang et al., 2021), deletion of proteases (Ahmad et al., 2019), and the introduction of chaperone factors (Zheng et al., 2019; Raschmanová et al., 2021). However, direct gene knockout in the genome can lead to the loss of specific functions within *P. pastoris*' metabolic pathways, potentially disrupting its overall metabolic network. In contrast, leveraging synthetic biology tools to modulate gene expression may offer a more efficient approach than traditional knockout or overexpression methods.

Gene expression regulation is fundamental to numerous cellular processes (de Nadal et al., 2011; Nielsen and Keasling, 2016). Currently, the primary tools for gene regulation in microorganisms are clustered regularly interspaced short palindromic repeats (CRISPR) systems. However, using CRISPR for gene activation or suppression often requires protein inactivation within the CRISPR system, the addition of activation or inhibitory domains, and careful selection of suitable sgRNA target sites. As a result, the CRISPR system is relatively complex and time-consuming. Furthermore, the application of CRISPR is also influenced by factors such as host cell acceptance, foreign protein expression efficiency, and target site selection accuracy, which makes the optimization process more cumbersome. In gene activation, additional transcriptional activators need to be introduced, while in gene suppression, inhibitory factors must be efficiently designed and delivered to ensure specific regulation. Therefore, despite its powerful gene regulation capabilities, the CRISPR system has high operational complexity and time costs (Casas-Mollano et al., 2020; Chen et al., 2020). In comparison, RNA interference (RNAi) targets RNA directly, influencing protein translation and offering a simpler method for gene regulation. RNAi is a gene silencing pathway activated by double-stranded RNA (dsRNA) (Drinnenberg et al., 2009), which is processed by the ribonuclease III (RNaseIII) enzyme Dicer into small interfering RNAs (siRNAs). Dicer is an enzyme responsible for cleaving double-stranded RNA into small siRNA fragments. These siRNAs then guide the Argonaute protein, which is involved in RNA cleavage, to target and cleave the transcripts, effectively silencing gene expression (Wang et al., 2019). The RNAi system, with its essential components—Dicer, Argonaute, and siRNAs—offers a more straightforward and flexible approach to gene silencing through simple plasmid transformation steps. This reduces time and effort, facilitating the rapid development of gene inhibition strategies across various strains (Crook et al., 2014).

This report details the first establishment of an RNAi system in *P. pastoris*. The hypothesis that such a system could be created was based on the observation that introducing the Argonaute protein and siRNA into *P. pastoris* could achieve RNAi-like effects. This suggested the potential presence of genes encoding Dicer-like proteins in the *P. pastoris* genome. The study successfully demonstrated single-gene (enhanced green fluorescent protein (*EGFP*)) and double-gene (*EGFP*/Histidine (*His*)) inhibition through the RNAi system by introducing hairpin RNAs. Genetically modified *P. pastoris* strains were used to modulate *EGFP* surface display, revealing that inhibiting the expression of endogenous genes Yeast AP-1-like transcription factor (*YAP1*) and Yeast Protease A (*YPS1*) reduced *EGFP* display levels by 83% and 48.8%, respectively. Conversely, inhibiting the expression of endogenous genes Proteinase B (*PRB1*) and Proteinase A (*PEP4*) resulted in increases in *EGFP* display levels by 33.8% and 26.5%, respectively. Furthermore, the RNAi system was applied to enhance the synthesis of the natural product 3-HP in *P. pastoris*.

In conclusion, the establishment of an RNAi system in *P. pastoris* and its successful application in enhancing both exogenous protein secretion and natural product synthesis demonstrate that RNAi is a powerful tool for gene expression regulation. This advancement significantly contributes to the development of *P. pastoris* cell factories, providing a promising platform for future biotechnological applications.

## 2 Materials and methods

### 2.1 Strains and culture conditions

The *Escherichia coli* Top10 strain was used for DNA manipulation. Cells were cultured in low-salt LB medium (1% tryptone, 0.5% NaCl, and 0.5% yeast extract) and selected with 25 μg/mL zeocin (Invitrogen) to confirm successful transformation of the bacterial plasmid. *P. pastoris* GS115 cells were used as host cells for protein expression. Transformants of *P. pastoris* were selected on YPDZ (1% yeast extract, 2% peptone, 2% glucose, 2% agar, and 25 μg/mL zeocin) plates after incubation for 2–3 days at 30°C.

For shake flask cultivation, the inoculum was cultured in 10 mL BMGY medium (1% yeast extract, 2% peptone, 1.34% YNB, 1% glycerol, 10% 1 mol/L pH 6.0 phosphate buffer) for 24 h to an initial OD600 of 0.5. The inoculum was then transferred to 25 mL BMMY (1% yeast extract, 2% peptone, 1.34% YNB, 1% methanol, 10% 1 mol/L pH 6.0 phosphate buffer), 10 mL semi-MM (0.67% YNB, 1% methanol), or 10 mL semi-MD (0.67% YNB, 1% glucose) culture medium for 120 h. Add 1% methanol or glucose every 24 h during cultivation to supplement the carbon source. (Control the initial OD600 of semi-MM and semi-MD media at 0.02. All cultures were conducted in BMMY medium unless otherwise specified.)

### 2.2 Construction method of hairpin RNA and dsRNA plasmids

Due to the repetitive sequences in both the hairpin RNA and dsRNA plasmids, all plasmid constructions required ligation using the DNA Ligase Kit (Thermo Scientific). For example, in the construction of pPICZA-*EGFP*-*His* (300 bp)-rad9-anti*EGFP*-*His* (300 bp), the *EGFP*-*His* (300 bp)-rad9 fragment was initially generated via overlap extension PCR, during which EcoRI and NotI restriction sites were introduced for the amplification of the *EGFP* (300 bp) and *His* (300 bp) sequences, respectively. The *EGFP*-*His* (300 bp)-rad9 fragment and the previously prepared plasmid pPICZA were then digested with EcoRI and NotI, followed by ligation with DNA ligase to construct the plasmid pPICZA-*EGFP*-*His* (300 bp)-rad9.

Next, the anti-*EGFP*-*His* (300 bp) DNA fragment was amplified, incorporating AgeI and NotI restriction sites for the amplification of the anti-*EGFP* (300 bp) and anti-*His* (300 bp) sequences, respectively. The anti-*EGFP*-*His* (300 bp) fragment was then digested with AgeI and NotI, as was the pPICZA-*EGFP*-*His* (300 bp)-rad9 plasmid. The two digested products were ligated using DNA ligase to generate the hairpin plasmid pPICZA-*EGFP*-*His* (300 bp)-rad9-anti*EGFP*-*His* (300 bp).

For the construction of the dsRNA plasmid, the process is exemplified through the construction of pPICZA-AOX1TT-PAOX1-*YAP1*-PAOX1-AOX1TT-*His4*. Using overlap extension PCR, the AOX1TT-PAOX1-*YAP1* fragment was amplified, incorporating EcoRI and BamHI restriction sites. The plasmid pPICZA-AOX1-AOX1TT-*His4* and the AOX1TT-PAOX1-*YAP1* fragment were then digested with EcoRI and BamHI, followed by ligation with DNA ligase to construct the dsRNA plasmid pPICZA-AOX1TT-PAOX1-*YAP1*-PAOX1-AOX1TT-*His4*.(The schematic diagrams of the construction process of the hairpin RNA plasmid and dsRNA plasmid can be found in Supplementary Figures 3, 4.)

### 2.3 Flow cytometry cell sorting

The dsRNA plasmid was electroporated into the GK3EA strain, and the cells were incubated on YPDZ plates at 30°C for 3 days. The resulting transformants were thoroughly mixed and co-cultured in BMGY medium for 36 h. The cells were then harvested, washed twice with 1.5 mL of PBS buffer, and resuspended in the same buffer. After dilution to an OD600 of approximately 0.5, the fluorescence intensity of the mixed library was analyzed using an S3eTM flow cytometer (Bio-Rad Laboratory, Inc.) with blue excitation light at 488 nm. The flow cytometry results were processed and analyzed using FlowJo software (TreeStar, Ashland, OR, United States). Approximately 100,000 cells were analyzed, and the top 1% of cells were sorted. The sorted cells were then plated onto YPDZ plates, incubated for 72 h, and 3–5 transformants were randomly selected for cultivation.

### 2.4 Reconstruction of the RNAi mechanism in *P. pastoris*

The Ago protein from *Saccharomyces castelli* was synthesized and codon-optimized for *P. pastoris* expression under the control of the constitutive PGAP promoter. The shRNA and dsRNA plasmids were constructed following the previously described procedure. Although the Dicer protein is encoded in the *P. pastoris* genome, the gene responsible for its expression remains unidentified, constituting the RNAi system in *P. pastoris*. To assess the functionality of the RNAi mechanism, we used a reporter system utilizing green fluorescent protein (*GFP*). After cultivation and culturing the strains, 200 µL of the yeast culture was centrifuged to remove the supernatant, and the cells were resuspended in 1 mL of PBS (pH 7.4) buffer. The suspension was centrifuged at 6000 rpm for 5 min and repeated three times. Finally, 1 mL of PBS buffer was added to resuspend the cells, and 200 µL of the suspension was used to measure the green fluorescence intensity in the yeast strains using a spectrophotometer (Biotek/Synergy H1/H1M) with 488 nm excitation and 520 nm emission.

### 2.5 qPCR (Quantitative PCR) was used to measure changes in gene transcription levels

Total RNA from *P. pastoris* was extracted using the hot phenol method. The extracted RNA samples were treated to remove genomic DNA (gDNA) and then reverse-transcribed using the PrimeScript™ RT Reagent Kit with gDNA Eraser. The concentration of the resulting cDNA was measured and diluted to 70 ng/μL. Quantitative real-time PCR (qRT-PCR) analysis was then performed using the SYBR^®^ Premix Ex Taq™ II (Tli RNaseH Plus) kit. The *GAPDH* gene in *P. pastoris* was used as a reference gene to measure the transcription levels of the *EGFP*, *HIS*, *PRB1*, *PEP4*, *YAP1*, *YPS1*, and *FAS1* genes. The data were analyzed using the 2^^-△△Ct^ method to quantify the relative changes in gene expression.

### 2.6 Detection of 3-hydroxypropionic acid

1.5 mL of the cultured broth is taken, centrifuged at 6000 rpm for 10 min, and the supernatant is collected. The supernatant is filtered through a 0.22 μm filter membrane to remove any remaining yeast. The concentration of 3-HP in the cultured liquid is measured using a Waters 1525 HPLC system. The treated sample is eluted through a 300 mm × 7.8 mm BioRad HPX-87H column, with a mobile phase of 0.5 mmol/L H_2_SO_4_, a flow rate of 0.4 mL/min, and an elution time of 25 min. UV detection is performed at 210 nm, and the column temperature is maintained at 60°C.

### 2.7 The *EGFP* protein is displayed on the surface of *P. pastoris*

The N-terminus of the *EGFP* gene is fused with the *α*-factor signal peptide, which guides the protein to the extracellular space. The C-terminus is fused with the GCW61 anchoring protein sequence, which anchors the *EGFP* protein to the cell wall. The *EGFP* expression cassette was constructed into a three-copy *EGFP* plasmid, pHKA-*EGFP*-Gcw61-3copy, using BglII and BamHI restriction enzymes. The plasmid pHKA-*EGFP*-Gcw61-3copy was linearized with the restriction enzyme Eam1105I and electroporated into GS115. Detailed DNA sequences are provided in the supplementary materials.

## 3 Results and discussion

### 3.1 The dicer protein expressed in *P. pastoris*

**FIGURE 1:**
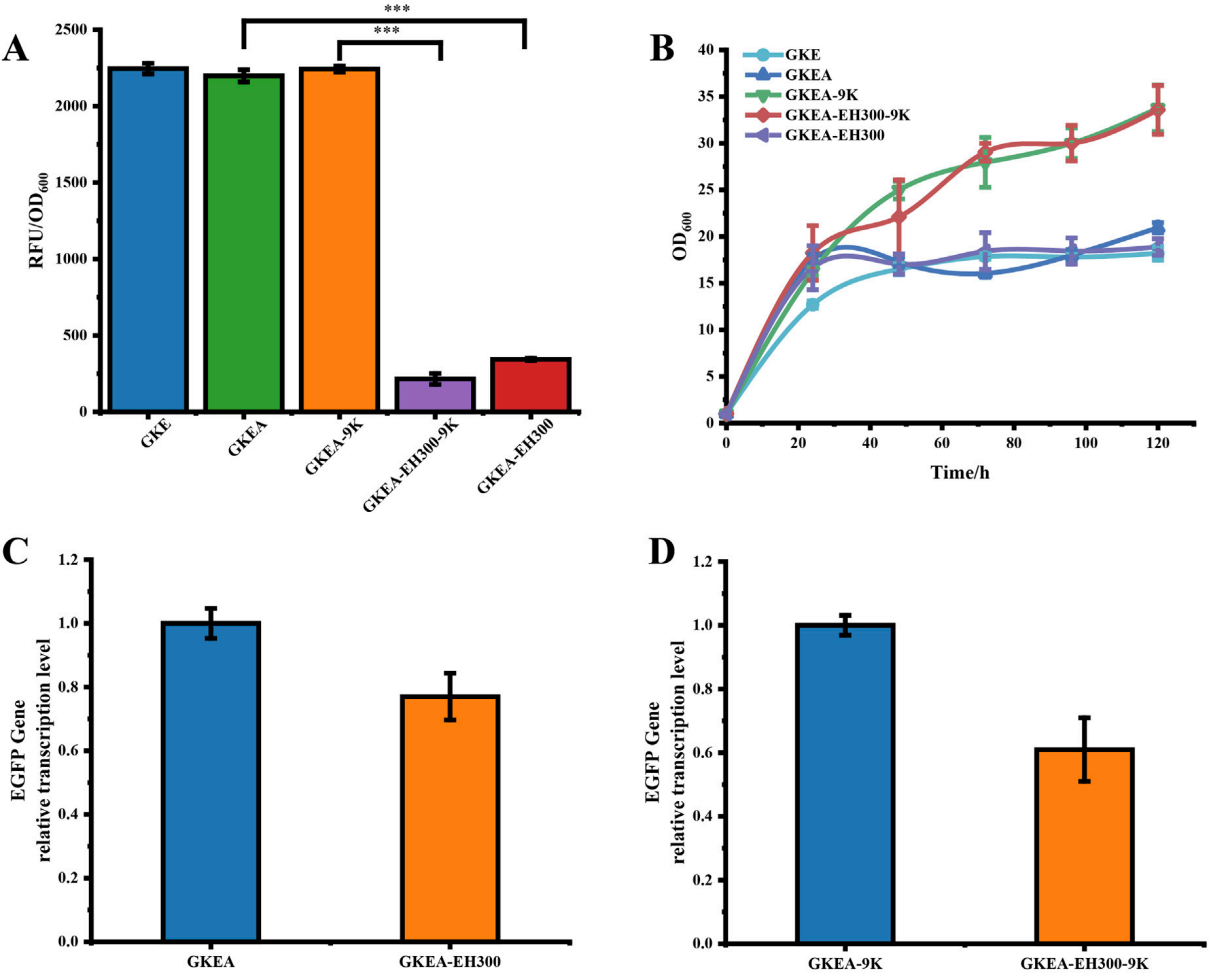
RNAi in single-gene silencing in *P. pastoris.* **(A)** RNAi-mediated silencing of *EGFP*. GKEA-EH300: No histidine complementation, *EGFP* inhibition; GKEA-9K: Histidine complementation only; GKEA-EH300-9K: Histidine complementation, dual inhibition of *EGFP/HIS*. **(B)** The growth condition of strains after *HIS* complementation. **(C)** The relative transcription level of *EGFP* in GKEA-EH300 at 72 h. **(D)** The relative transcription level of *EGFP* in GKEA-EH300-9K at 72 h. The error bars represent the standard error of the mean. The sample size for each boxplot is n = 3, and statistical significance was calculated using one-way ANOVA. NS indicates no significant difference; *P < 0.05; **P < 0.01; ***P < 0.001.

RNA interference (RNAi) is a gene silencing mechanism activated by double-stranded RNA (dsRNA). The RNAi pathway in budding yeast involves three essential components: dsRNA, the specific endoribonuclease Dicer, and the binding protein Ago. The previous study has reported the loss of RNAi functionality in most budding yeast species, although some species retain the Ago protein (Drinnenberg et al., 2009). In *Saccharomyces cerevisiae*, neither the Dicer protein, the Ago protein, nor double-stranded RNA has been detected. Through genome alignment, we identified an endogenous protein in *P. pastoris* that may function similarly to the Dicer protein in *Saccharomyces castellii*, named RNAase III (PAS_chr4_0127). Additionally, by introducing only the Ago protein into *P. pastoris*, we observed that the RNAi system can function normally in *P. pastoris* (Figure 1A). This suggests the presence of a Dicer gene in *P. pastoris*, although the specific gene encoding the Dicer protein has not yet been identified in this study.

### 3.2 RNAi-mediated silencing of single genes in *P. pastoris*

Hairpin RNAs, double-stranded RNAs (dsRNAs), and full-length antisense RNAs have been shown to serve as substrates for Dicer in generating small interfering RNAs (siRNAs). These siRNA duplexes mediate mRNA degradation in collaboration with Argonaute proteins, effectively suppressing the expression of target genes (Chen et al., 2020). Among these, hairpin RNA is particularly efficient due to its higher recognition affinity by Dicer and greater RNA interference (RNAi) activation capability, making it the preferred precursor for single-gene suppression (Liu et al., 2002).

To evaluate the RNAi system in *P. pastoris*, A plasmid, pPICZA-*EGFP*-*HIS4*300-rad9-anti*EGFP*-*HIS4*300, was constructed containing fragments of the *EGFP* and *HIS* genes, each featuring a 300 bp forward sequence followed by a 300 bp inverted repeat sequence. A linker sequence was added between the inverted repeat sequences at both ends to facilitate the folding back of the repeat sequences after transcription and form the shRNA structure. Experimental results revealed that the histidine-deficient GS115 strain of *P. pastoris* exhibited partial growth inhibition in the GKEA and GKEA-EH300 strains, with the highest OD600 value reaching approximately 20 (Figure 1B). Growth was restored upon complementation of the *HIS* gene, confirming the dependency on histidine.

In fluorescence assays, the GKEA strain (positive control) showed a significantly higher RFU/OD600 of 2198.12. In contrast, the GKEA-EH300 strain exhibited a dramatic reduction in relative fluorescence intensity, with an RFU/OD600 of 343. Similarly, after histidine complementation, the GKEA-9K positive control strain maintained a high RFU/OD600 value of 2242, whereas the GKEA-EH300-9K strain demonstrated a substantially reduced RFU/OD600 value of 215, consistent with prior observations (Figure 1A).

To confirm that the reduced fluorescence intensity was attributable to the RNAi mechanism, transcriptional analysis of the *EGFP* gene was performed for the GKEA-EH300 and GKEA-EH300-9K strains. Results indicated a clear reduction in *EGFP* transcription levels (Figures 1C, D). These findings collectively demonstrate that the RNAi system is functional in *P. pastoris*, effectively mediating gene silencing.

### 3.3 RNAi-based negative modulation of dual genes expression in *P. pastoris*

Figure 1B demonstrates that despite the repression of the *HIS* gene, the growth of the GKEA-EH300-9K strain was not significantly impaired, likely due to the abundant carbon sources in BMMY medium, which supported normal growth. To further investigate the effect of simultaneous repression of both gene, the GKEA-EH300-9K strain was cultured under semi-MM and semi-MD culture conditions. After 96 h of fermentation, growth conditions and relative fluorescence intensities were evaluated.

**FIGURE 2:**
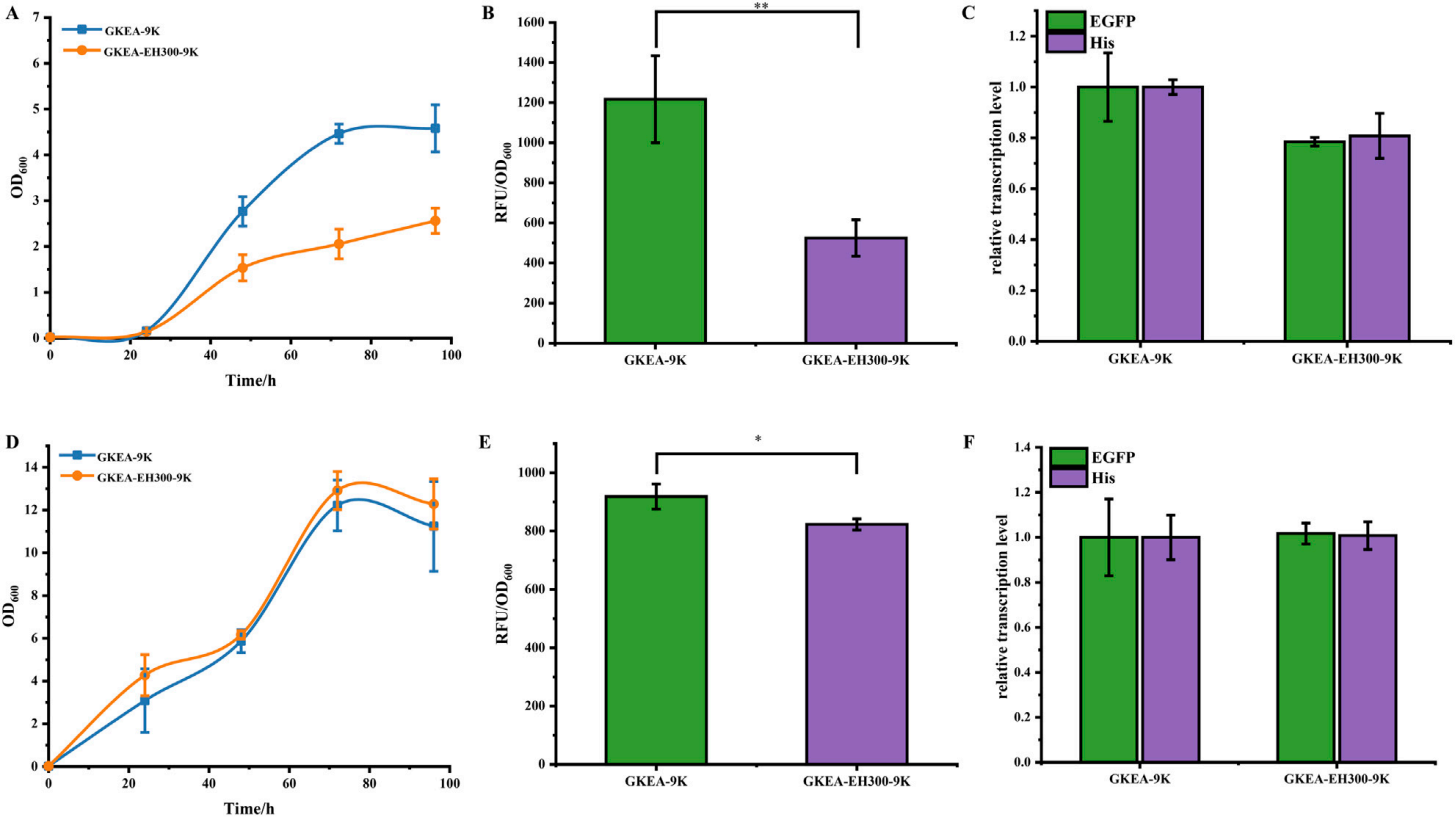
RNAi in dual gene silencing in *P. pastoris.* **(A)** RNAi-mediated histidine silencing in GKEA-EH300-9K in semi-MM medium. **(B)** RNAi-mediated *EGFP* silencing in GKEA-EH300-9K in semi-MM medium. **(C)** Transcription levels of *EGFP* and *HIS* genes in GKEA-EH300-9K under semi-MM medium. **(D)** RNAi-mediated silencing of *HIS* in GKEA-EH300-9K in semi-MD medium. **(E)** RNAi-mediated silencing of *EGFP* in GKEA-EH300-9K in semi-MD medium. **(F)** Transcription levels of *EGFP* and *HIS* genes in GKEA-EH300-9K under semi-MD medium. The error bars represent the standard error of the mean. The sample size for each boxplot is n = 3, and statistical significance was calculated using one-way ANOVA. NS indicates no significant difference; *P < 0.05; **P < 0.01; ***P < 0.001.

The results showed that under semi-MM conditions, the growth of the GKEA-EH300-9K strain was significantly inhibited compared to the GKEA-9K strain (Figure 2A). However, under semi-MD medium, the growth of GKEA-9K and GKEA-EH300-9K strains was similar (Figure 2D). These observations suggest that the RNAi system effectively suppressed the expression of the *HIS* gene, leading to growth limitation of the GKEA-EH300-9K strain in semi-MM medium.

Further evaluation of the relative fluorescence intensity of all strains under semi-MM and semi-MD conditions at 72 h of cultivation was conducted. In the 72-hour cultivation, under semi-MM conditions, the relative fluorescence intensity of the GKEA-EH300-9K strain decreased by 57% compared to the GKEA-9K strain (Figure 2B). In contrast, under semi-MD conditions, there was no significant difference in relative fluorescence intensity among the strains (Figure 2E). This result is attributed to the inhibitory effect of the methanol-induced AOX1 promoter under glucose-rich conditions, which suppressed the expression of the EH300 plasmid, thus eliminating the inhibitory effect under semi-MD conditions. Additionally, transcriptional analysis provided further insights. Under semi-MM medium, the transcription levels of *EGFP* and *HIS* genes in the GKEA-EH300-9K strain decreased by approximately 20%. In contrast, under semi-MD conditions, there were no significant changes in the transcription levels of the GKEA-EH300-9K strain (Figures 2C, F).

These findings collectively confirm that under semi-MM conditions, the RNAi system successfully interfered with the expression of both target genes in *P. pastoris*, as evidenced by growth inhibition and reduced fluorescence intensity.

### 3.4 Reducing the production of *EGFP* in *P. pastoris* by RNAi

By leveraging RNAi to disrupt the expression of key genes in the exogenous protein expression pathway of *P. pastoris*, it is possible to regulate exogenous protein expression levels. The transcription factor *YAP1* and the protease *YPS1* play critical roles in this pathway. *YAP1* expression mitigates cellular damage caused by reactive oxygen species (ROS), thereby enhancing protein expression (Delic et al., 2014). Conversely, studies have shown that knocking out the *YPS1* gene reduces the degradation of exogenous proteins (Sazonova et al., 2013; Wu et al., 2013).

In this study, *EGFP* was used as a reporter protein, and double-stranded RNA (dsRNA) targeting the *YAP1* and *YPS1* genes was introduced into the genome of the GK3EA yeast strain to establish an RNAi system.

**FIGURE 3:**
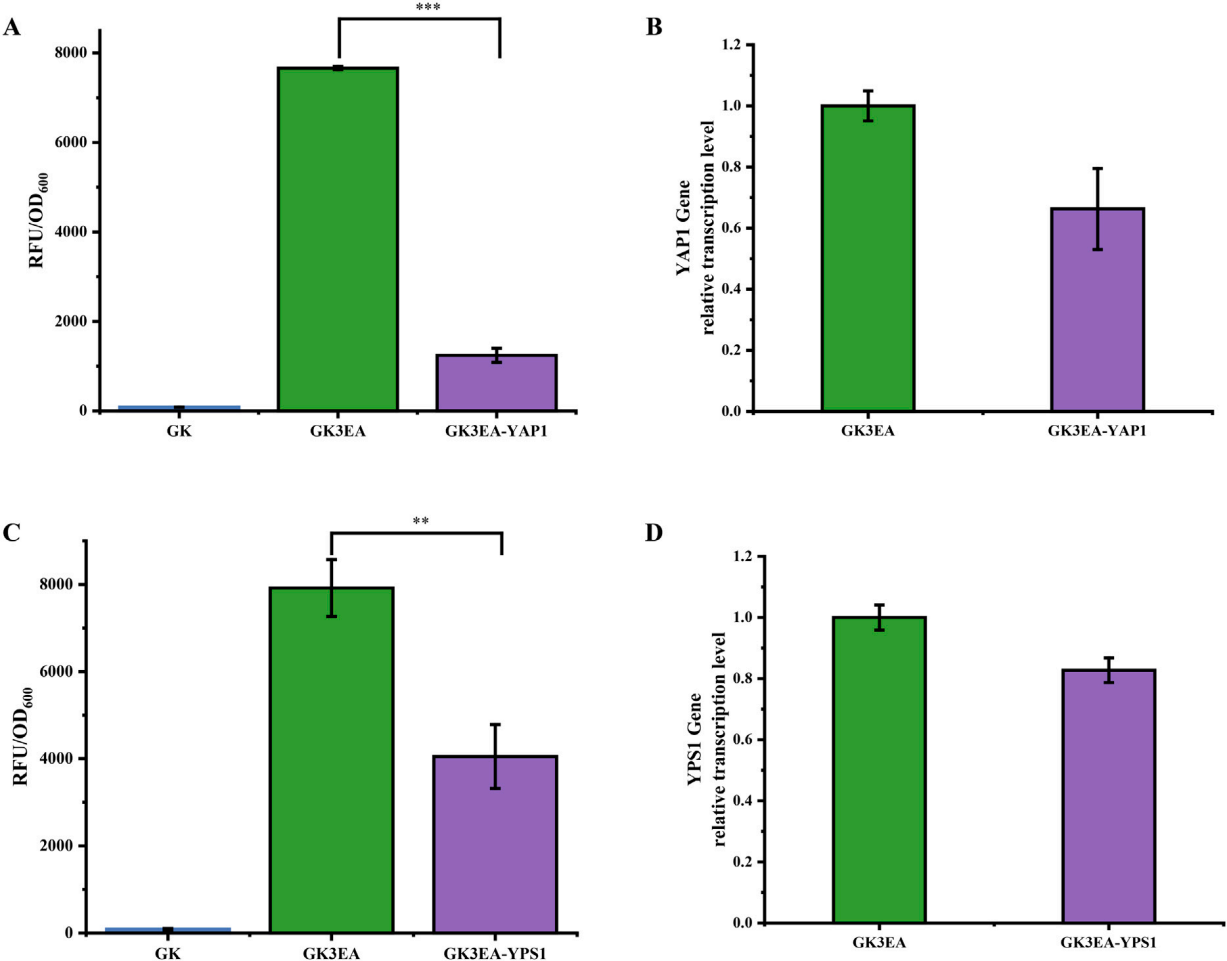
Reduction of *EGFP* surface display ability in *P. pastoris* through RNAi. **(A)** The relative fluorescence intensity of the GK3EA-*YAP1* at 72 h. **(B)** Relative transcription level of *YAP1* in the GK3EA-*YAP1* at 72 h **(C)**The relative fluorescence intensity of the GK3EA-*YPS1* strain at 72 h. **(D)** Relative transcription level of *YPS1* in the GKEA-*YPS1* at 72 h. The error bars represent the standard error of the mean. The sample size for each boxplot is n = 3, and statistical significance was calculated using one-way ANOVA. NS indicates no significant difference; *P < 0.05; **P < 0.01; ***P < 0.001.

The results revealed varying degrees of reduction in *EGFP* expression after integrating *YAP1* and *YPS1* dsRNAs. After 72 h of fermentation, the ability of yeast cells containing three copies of the (*EGFP*) gene to display *EGFP* was reduced by 83% in strains with *YAP1* dsRNA integration. (Figure 3A). Similarly, a 48.8% reduction in this ability was observed in strains with *YPS1* dsRNA integration after 72 h of fermentation (Figure 3B). Flow cytometric analysis of the GK3EA-*YAP1* and GK3EA-*YPS1* strains confirmed that the mean fluorescence intensities were lower than those of the GK3EA strain when 100,000 cells were analyzed, indicating successful RNAi-mediated suppression of *YAP1* and *YPS1* expression. This suppression reduced the ability to display *EGFP* on the cell surface (Supplementary Figure 1).

To further confirm that the reduction in fluorescence intensity was due to RNAi action, transcription level assays were conducted on the GK3EA-*YAP1* and GK3EA-*YPS1* strains. Compared to the GK3EA strain, transcription levels of the *YAP1* gene in GK3EA-*YAP1* and the *YPS1* gene in GK3EA-*YPS1* were significantly decreased (Figures 3C, D).

These findings demonstrate that the RNAi system can effectively interfere with the expression of endogenous genes involved in the exogenous protein expression pathway of *P. pastoris*. This approach shows promise for fine-tuning the expression of exogenous proteins by modulating the associated endogenous genes.

### 3.5 Increasing the production of *EGFP* in *P. pastoris* by RNAi

The genes *PRB1* and *PEP4* encode vacuolar proteinase B and vacuolar proteinase A (Moehle et al., 1987; Woolford et al., 1986; Marsalek et al., 2019), respectively, both of which play significant roles in protein degradation. The previous study has reported that deleting these genes can enhance exogenous protein expression (Marsalek et al., 2019). Building on this insight, an RNAi system was developed to increase *EGFP* expression by integrating dsRNAs targeting *PRB1* and *PEP4* into GK3EA yeast cells.

**FIGURE 4:**
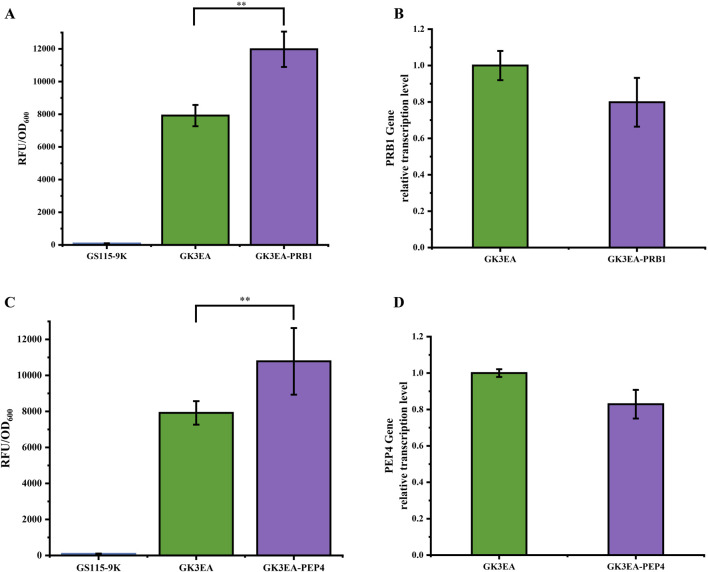
Enhancing the surface display ability of *EGFP* in *P. pastoris* through RNAi. **(A)** Relative fluorescence intensity of GK3EA-PRB at 72 h. **(B)** Relative transcription level of *PRB1* in the GK3EA-*PRB1* at 72 h. **(C)** Relative fluorescence intensity of GK3EA-*PEP4* at 72 h. **(D)** Relative transcription level of *PEP4* in the GK3EA-*PEP4* at 72 h. The error bars represent the standard error of the mean. The sample size for each boxplot is n = 3, and statistical significance was calculated using one-way ANOVA. NS indicates no significant difference; *P < 0.05; **P < 0.01; ***P < 0.001.

The results demonstrated notable increases in *EGFP* expression in yeast strains following the integration of dsRNAs targeting *PRB1* and *PEP4*. After 72 h of cultivation, yeast strains with *PRB1* dsRNA integration showed a 33.8% increase in the ability of yeast cells containing three copies of the gene to display *EGFP* (Figure 4A). Similarly, strains with *PEP4* dsRNA integration exhibited a 26.5% increase in *EGFP* surface display (Figure 4C). The flow cytometry histograms of the GK3EA-*PRB1* strain were consistent with the cultivation results, further corroborating these findings (Supplementary Figure 2).

Transcriptional analysis revealed a measurable decrease in the transcription levels of the *PRB1* gene in the GK3EA-*PRB1* strain and the *PEP4* gene in the GK3EA-*PEP4* strain compared to the GK3EA strain. These results confirm the successful suppression of *PRB1* and *PEP4* expression by the RNAi system (Figures 4B, D).

This study highlights the effectiveness of employing an RNAi system to repress endogenous genes that influence protein degradation pathways. By targeting *PRB1* and *PEP4*, this approach successfully enhances exogenous protein expression, providing a powerful strategy for optimizing global protein production in *P. pastoris*.

### 3.6 Enhancing 3-hydroxypropionic acid production in *P. pastoris* via RNAi

Building on the successful modulation of *EGFP* expression in *P. pastoris*, the RNAi system was further applied to regulate key metabolic pathways for 3-hydroxypropionic acid (3-HP) production, demonstrating the versatility of the RNAi system in both protein expression and metabolic engineering. By targeting fatty acid synthesis, we aimed to enhance malonyl-CoA availability, a crucial precursor for 3-HP biosynthesis, thus expanding the utility of RNAi technology beyond exogenous protein production to valuable natural product synthesis.

3-HP is a highly versatile platform chemical, extensively utilized in the synthesis of acrylic acid, methyl methacrylate, propiolactone, and malonic acid (Wu et al., 2023; Qin et al., 2024). Recognizing its substantial industrial value, the U.S. Department of Energy has identified 3-HP as one of the most promising platform chemicals (Qin et al., 2024).

The use of *P. pastoris* as a microbial cell factory for 3-HP production offers significant potential due to its favorable attributes for industrial bioprocessing. In this study, the synthetic pathway genes for 3-HP production were successfully integrated into the *P. pastoris* GS115 strain, resulting in the development of a 3-HP-producing strain (HP strain). However, achieving optimal yields of 3-HP remains a formidable challenge, necessitating further advancements in strain engineering and process optimization to fully harness its industrial potential.

**FIGURE 5:**
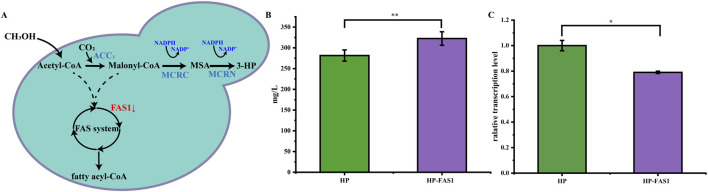
Enhancing 3-HP biosynthesis in *P. pastoris* through RNAi. **(A)** Schematic diagram of 3-HP production via methanol metabolism in *P. pastoris*. **(B)** The 3-HP production of the HP-FAS1 strain at 72 h. **(C)** The transcription level of the FAS1 gene in the HP-FAS1. The error bars represent the standard error of the mean. The sample size for each boxplot is n = 3, and statistical significance was calculated using one-way ANOVA. NS indicates no significant difference; *P < 0.05; **P < 0.01; ***P < 0.001.

To enhance the conversion efficiency of malonyl-CoA to 3-hydroxypropionic acid (3-HP), this study utilized RNAi technology to suppress fatty acid synthesis, thereby increasing malonyl-CoA availability. The HP strain was further engineered by introducing the Ago plasmid and a bidirectional promoter plasmid containing an FAS1 gene fragment, resulting in the development of the HP-FAS1 strain (Figure 5A).

By employing this RNAi strategy, the 3-HP yield in the HP-FAS1 strain increased significantly, rising from 281 mg/L to 322 mg/L (Figure 5B). Furthermore, transcriptional analysis revealed a 20% reduction in FAS1 expression in the HP-FAS1 strain (Figure 5C), underscoring the effective implementation of RNAi technology to enhance 3-HP biosynthesis in *P. pastoris*. This study not only underscores the transformative impact of RNAi on improving 3-HP production but also serves as a compelling example of employing RNAi systems in *P. pastoris* to boost the biosynthesis of other valuable natural products. These findings provide critical insights into the application of advanced genetic engineering techniques in industrial microorganisms, paving the way for further innovations in biomanufacturing.

In summary, RNAi technology enhances the synthesis efficiency of target products (such as 3-HP) by inhibiting specific metabolic pathways, reducing resource wastage during the production process, and thus lowering production costs. Additionally, RNAi technology does not rely on complex gene editing tools, making it simple to operate and cost-effective, which is suitable for large-scale production. By improving the efficiency of metabolic flux, it can significantly increase product yield, thereby boosting the economic benefits of the production process. Furthermore, the high tunability and flexibility of RNAi technology enable its broad application across different microbial production platforms, further enhancing its scalability and adaptability in industrial production. Therefore, RNAi technology provides an economically efficient and scalable solution for large-scale *P*. *pastoris* production, with strong market potential.

## 4 Conclusion

With the rapid advancement of synthetic biology tools and the inherent adaptability of *P. pastoris*, this yeast has quickly become one of the most attractive hosts for heterologous protein production. In microbial production processes, achieving optimal yields of products or synthetic proteins often necessitates fine-tuning the expression of key enzymes within specific metabolic pathways. The RNAi system, being a simple and efficient tool, is particularly suited for this task, offering an innovative solution for high-throughput screening and other metabolic engineering applications, thus facilitating the development of engineered *P. pastoris* strains.

RNAi studies in yeast began with *Saccharomyces cerevisiae* model strains. Early work by P. Bartel and colleagues introduced Dicer and Argonaute proteins from *Saccharomyces castellii* into brewing yeast to reconstruct the RNAi system. This was used to silence endogenous retrotransposons, successfully integrating RNAi tools into budding yeast research (Drinnenberg et al., 2009). Subsequently, S. Alper and colleagues outlined design principles for constructing hairpin RNA expression cassettes in yeast, leveraging RNA interference to enhance erythromycin production (Crook et al., 2014). Building on these efforts, Petranovic and colleagues utilized droplet microfluidics screening combined with experimental validation to identify RNAi targets that enhance recombinant protein production in *S. cerevisiae* (Wang et al., 2019). Inspired by these advancements, we aimed to develop an RNAi system for *P. pastoris* to streamline the design-build-test cycle while reducing costs.

In this study, we successfully established an RNAi system in *P. pastoris* capable of silencing single or double genes. Using this system, we identified effective genetic targets and implemented gene inhibition strategies to modulate the surface display levels of exogenous *EGFP*, either enhancing or reducing its expression. Additionally, to demonstrate the versatility of this system, we applied it to suppress fatty acid synthesis, resulting in increased malonyl-CoA availability and improved 3-HP production.

The expansion of RNAi systems represents a significant milestone in the metabolic engineering of *P. pastoris*. This technology provides an efficient and convenient method for regulating gene expression, particularly for genes requiring precise modulation. By integrating RNAi with high-throughput screening methods, further potential genetic targets in *P. pastoris* can be uncovered, significantly enhancing its practical applications as a versatile cell factory. It is important to note that the application of this method is not limited to *P. pastoris* but can also be extended to other microbial systems, further advancing the use of RNAi technology in microbial metabolic engineering. Future research directions could include applying RNAi systems to other types of microorganisms to optimize their production capacity and further improving RNAi technology for greater effectiveness and precision.

Although RNAi technology has demonstrated strong potential in gene suppression, its potential limitations in application also need to be addressed. First, gene copy number significantly affects the efficiency of RNAi suppression. In some cases, a higher gene copy number may reduce the effectiveness of the RNAi system, especially when excessive gene copies are expressed in the cell, which may require more RNAi reagents to achieve effective suppression. Additionally, RNAi technology may have “off-target effects” during the suppression of certain genes, meaning unrelated genes or regulatory elements may be suppressed, leading to some side effects. Furthermore, the persistence of RNAi suppression may also be limited, particularly in long-term cultures or large-scale production, where the effect of RNAi may gradually weaken. Therefore, future research should thoroughly explore these limitations and optimize the RNAi system to improve its application effectiveness and scalability in different microbial systems.

In summary, we developed a robust RNAi-based gene expression regulation toolkit for *P. pastoris*, enabling both single- and double-gene suppression. This toolkit was successfully used to fine-tune endogenous gene expression, optimizing the surface display of *EGFP* and improving 3-HP synthesis. Our work highlights the potential of RNAi as a powerful resource for advancing the efficiency and versatility of *P. pastoris* as a cell factory for industrial applications.

## Data Availability

The raw data supporting the conclusions of this article will be made available by the authors, without undue reservation.
