# Supplementary material for: Establishment and application of an RNAi system in Pichia pastoris
Source: Front Bioeng Biotechnol. 2025 Mar 5;13:1548187. doi: 10.3389/fbioe.2025.1548187 (PMC11919887; doi:10.3389/fbioe.2025.1548187)
Supplement: Supplementary file 1 [file DataSheet1.pdf]

Supplementary Materials for

Establishment and application of an RNAi system in *Pichia pastoris*

Supplementary Table S1 Plasmids constructed in this study

Plasmids	Genotype	Source	Description
pGAPZA_Ago1_lac_cre_lox p_HIS4	zeocin, pGAP	This study	Components of the RNAi System
pTEFZA_EGFP_lac_cre_lox p_HIS4	zeocin, pTEF	This study	For the intracellular expression of EGFP in *P. pastoris*
pHKA-EGFP-Gcw61-3copy	Kana, pAOX1	This study	Surface display expression of EGFP in *P. pastoris*
pPICZA-EGFP-HIS4300-rad 9-antiEGFP-HIS4300	zeocin, pAOX1	This study	
pPICZA-AOXTT-pAOX1-Y PS1-pAOX1-AOXTT-His4	zeocin, pAOX1	This study	
pPICZA -AOXTT-pAOX1-YAP1-pA OX1-AOXTT-His4	zeocin, pAOX1	This study	
pPICZA -AOXTT-pAOX1-PRB1-pA OX1-AOXTT-His4	zeocin, pAOX1	This study	
pPICZA	zeocin, pAOX1	This study	

-AOXTT-pAOX1-PEP4-pAOX1-AOXTT-His4			
pMD18T-PNSIV9-AOXTT-pAOX1-FAS1-pAOX1-AOXTT	Amp, pAOX1	This study	Enhancing 3-hydroxypropionic acid production in *P. pastoris*

Supplementary Table S2 List of oligonucleotides used in this study

Primer	Sequence (5'-3')
Ago-S	ATGGATTACAAGGATGATGACGATAAGTCAT
Ago-A	TCATATGTAGTACATGATGTCAGTGACATTCTTATGAAC
YAP1-S	ATGAGTGACGTGGTAAACAAGAGAGCGG
YAP-A	CGAGGAGCTCTTCACCGATTCGGCCGTCAAC
YPS1-S	ACTCATCACTTCCAGAGCTGAATATTCCAATA
YPS1-A	GATATACATGATGAGAAGATCGCCGAATGGGATCCCATCA
PRB1-S	ATGATATTTGACGGTACTACGATGTCAATT
PRB1-A	ATGCAATTGCGTCATTCCGTTGGATTGGCTATC
PEP4-S	GATATACATGATGAGAAGATCGCCGAATGGGATCCCATCA
PEP4-A	ATGATATTTGACGGTACTACGATGTCAATT
FAS1-S	TGTAGTCCTCTAACACCAATGTAGAACATGAAT

FAS1-A	ATTGAAAATCAAAGAAAACTTAATCAAGAACTACTACCT

Supplementary Table S3 Strains used in this study

Strain	Genotype	Source
GS115-*ΔKu70-EGFP (GKE)*	*PTEF1::EGFP, ΔKu70*	This study
GS115-*ΔKu70-EGFP-Ago (GKEA)*	*PTEF1::EGFP, PGAP::Ago, ΔKu70*	This study
GS115-*ΔKu70-EGFP-Ago*-PPIC9K *(GKEA-9K)*	*HIS, PTEF1::EGFP, PGAP::Ago, ΔKu70*	This study
GS115-*ΔKu70-EGFP-Ago*-EH300 *(GKEA-EH300)*	*PTEF1::EGFP, PGAP::Ago, ΔKu70, PAOX1::EH300*	This study
GS115-*ΔKu70-EGFP-Ago*-EH300-PPIC9K *(GKEA-EH300-9K)*	*HIS, PTEF1::EGFP, PGAP::Ago, ΔKu70, PAOX1::EH300*	This study
GS115-ΔKu70-3copy-EGFP-Ago (GK3EA)	*PGAP::Ago,HIS::3copy-EGFP, ΔKu70*	This study
GS115-*ΔKu70*-3copy-*EGFP-Ago-YAP1 (GK3EA-YAP1)*	*PGAP::Ago,HIS::YAP1,3copy-EGFP, ΔKu70*	This study
GS115-*ΔKu70*-3copy-*EGFP-Ago-YPS1 (GK3EA-YPS1)*	*PGAP::Ago,HIS::YPS1,3copy-EGFP, ΔKu70*	This study
GS115-*ΔKu70*-3copy-*EGFP-Ago-PRB1 (GK3EA-PRB1)*	*PGAP::Ago,HIS::PRB1,3copy-EGFP, ΔKu70*	This study
GS115-*ΔKu70*-3copy-*EGFP-Ago-PEP4*	*PGAP::Ago,HIS::PEP4,3copy-EGFP,*	This study

(GK3EA-PEP4)	*ΔKu70*	
GS115-MCRC-MCRN (HP)	*PNSII4: MCRC, MCRN*	This study
GS115-MCRC-MCRN-Ago-AOXTT-pAO	*PNSII4: MCRC, MCRN PNSI6: Ago*	This study
X1-FAS1-pAOX1-AOXTT	*PNSIV9:*AOXTT-pAOX1-FAS1-pAO	
（HP-FAS1）	X1-AOXTT	

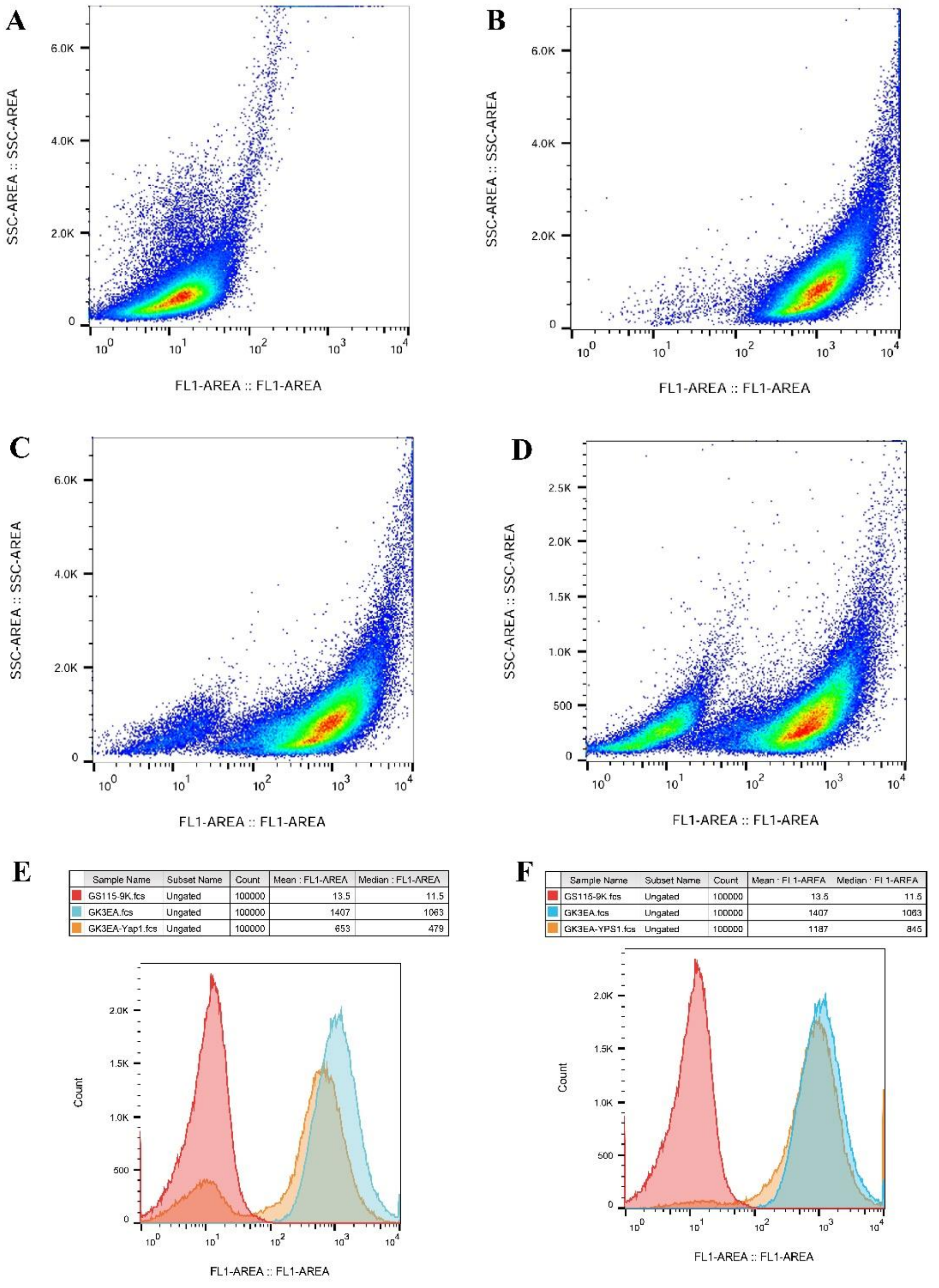

Supplementary Figure 1.

(A) Density graph of GS115-9K strain cultivation at 36 hours

(B) Density graph of GK3EA strain cultivation at 36 hours

(C) Density graph of GK3EA-YAP1 strain cultivation at 36 hours

(D) Density graph of GK3EA-YPS1 strain cultivation at 36 hours

(E) Histogram of cultivation of GK3EA-YAP1 strain at 36 hours

(F) Histogram of cultivation of GK3EA-YPS1 strain at 36 hours

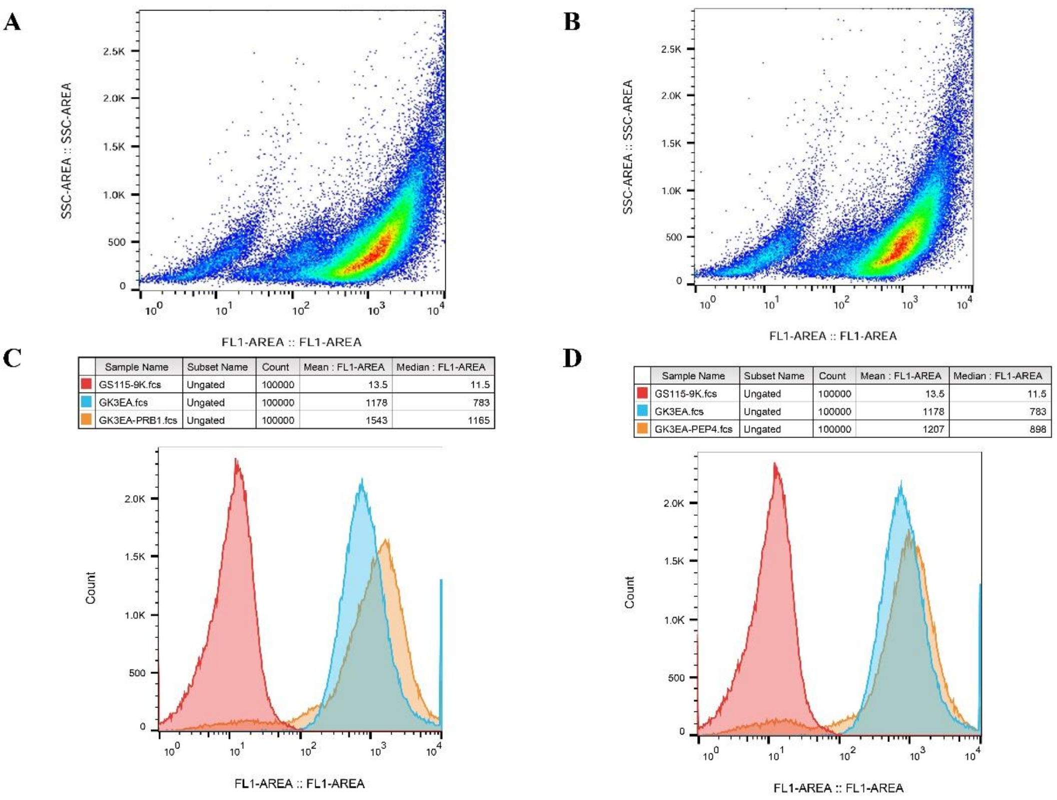

Supplementary Figure 2.

(A) Density graph of GK3EA-PRB1 strain cultivation at 36 hours

(B) Density graph of GK3EA-PEP4 strain cultivation at 36 hours

(C) Histogram of cultivation of GK3EA-PRB1 strain at 36 hours

(D) Histogram of cultivation of GK3EA-PEP4 strain at 36 hours

Schematic diagram of the construction of hairpin RNA and dsRNA plasmids

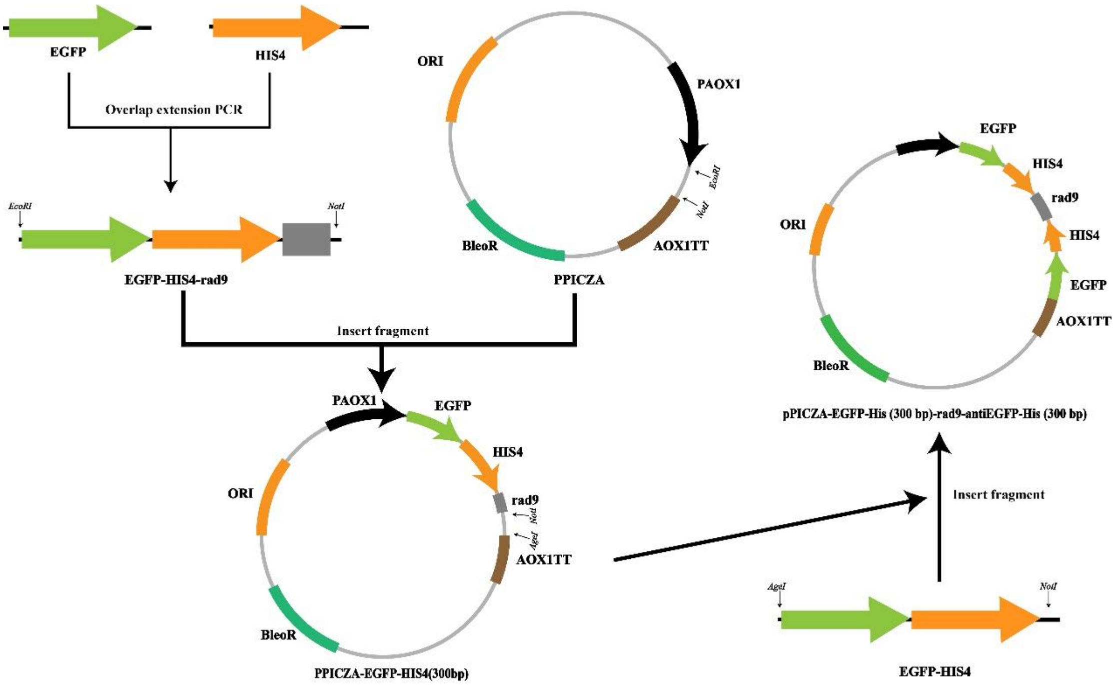

Supplementary Figure 3: The diagram illustrates the construction of hairpin plasmid

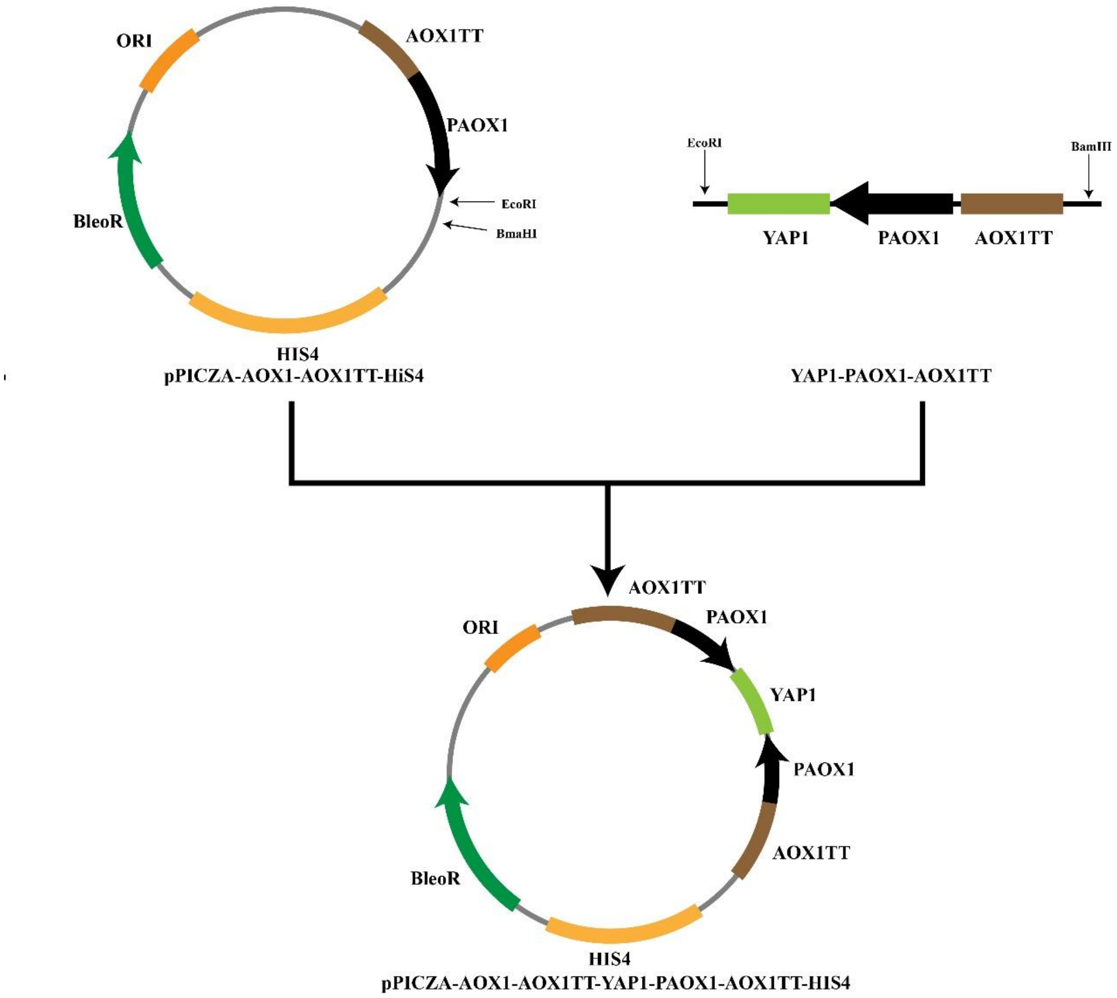

Supplementary Figure 4: The diagram illustrates the construction of dsRNA plasmid

The OD600 of the strains with enhanced or reduced surface display ability of EGFP in *P. pastoris* at 72 hours of cultivation

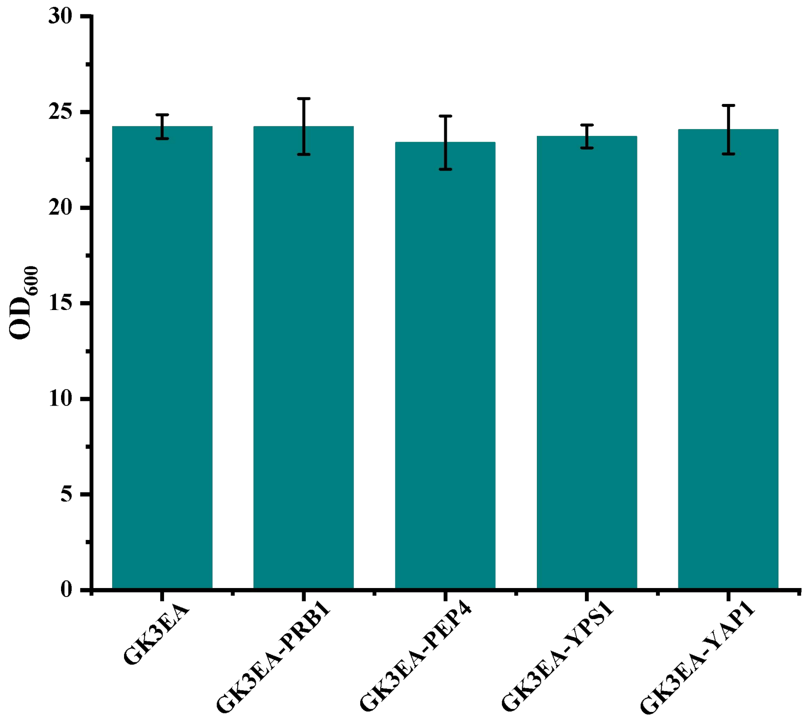

Supplementary Figure 5: The OD600 of each strain at 72 hours of cultivation, with RNAi-mediated improvement in EGFP surface display ability.

Note: Compared to the control strain GK3EA, the OD600 of the GK3EA-PRB1, GK3EA-YAP1, GK3EA-PEP4, and GK3EA-YPS1 strains showed minimal changes, indicating that the changes in RFU/OD are not due to variations in OD.

RNAi plasmids and related gene sequences：

DNA sequence of the Ago gene (codon-optimized for *P. pastoris*):

ATGGATTACAAGGATGATGACGATAAGTCATCCAATTCGGAGGAGAACAGTCAAGTTC
CTCCCCTTGATGCCACCGCTGCTGCAACTAAACCAAAAAAGGCCAAGAAACCAAAGG
TTAAGAAGCCAAAGGATTCCGCTGAGGCATCTTCATCACCAGCTGCTGAGGGCACTGC
TGAAGCCAAGCCAAAAAAGGCTAAGAAATCAAAGACTAAGAAGTCAAAGGAGTCTG
CCGAGGTATCGCCAGCTCCAGCTGATGAAACTACTTCTGCTGGTGTAGATGCTAAACCT
AAGAAGGCTAAAAAGTCAAAGGTCAAGAAGCCAAAGGATTCTACTGAATCATCTCCT
GCTCCAAGTAATGAACCTCCAGCCGCCGAAGTTGCCGCCGAAGATGCAAAGTCTAAA
AAGGTTAAGAAACCAAAGGCCAAGAAACCAAAGGAATCTACTGAATCATCTCCTGCTC
CAGGTCAGGAAGCCGCTGCTACTGAAGGTGCCACAGAAGATAAACCTAAGAAGGTTA
AGAAATCAAAGGCTAAGAAGGCAAAGGAATCTGTTGAATCATCTCCTGCCGCCACTGA
ATCTGTCTCTGAGAAGACTGCAAAGAAATCTAAGAAGCCAAAGGCTAAGAAATCTACT
TCTCCTGAAACAACTGAAGAAATAACTGAAGAATCTACCGAATCAAAGGAAAAGAAG
ACAAAGACAAAGAAACCAAAGGAAAAGAAGTCATCTCCATCTACTGCCACTTCGACT
GCTGCTTCAAAACCAGTTACTTCCATTGCGGGAGTCACCATTCCAGGCAAGACTTTTGA
TTTGACAGATGTTCCTCCATCTAAACCTGCTCCAAAGATGGTCCCGGAAGCCTATAAAT
TACAAACTAGAGTGGATTACGGTACTAAGGGTACCAAAGTGGACGTCTTGACTAATCAT
ATACTACTATCTGTTGGTGATGATGTCCCTCAAGATGAAAGAGCCTCTCAATTGGATCCA
TGGTGGAAATCTGCATTTGTATATACCTATAACATTACTTTTGCCGTTCCACAGAGTAATT
CACCACGTAAAGGTCCAGCGCCTGCTTTATCCAAACCAAAGAAATACGAATTGGTAGA
ATCTTTGTTTCACGAGGATGAAACCTTGTTCAAATATAAAGATCGTATCTCCTTCAATGG
TGAAGATACTCTATACTCTCATGTCCCATTGGAGGAATTCACTTTATTTGATGGTTGTTG
GGATGTCAGTAACAAGCAAAAGAAAAGAAGACAAGAAGTTGTTGGTCTCAACAGTAG
AGCCAAGGAAATTAATGACTTGGCTGCTCAAGTTACTTTAAAATTTGCTGATAAAGTCC
CATTGGGTGATATTTATAAGGCTACCACTTCAAAGGATCCAGAAGAACAAGAAAATAA
GATGGCTAACGCTGATAAGGTTGCTTTATTGTCTTTGATGGGTGTCAAATTCTTAAACAC
AAAGGAACAAATCTTCCAACTTAACGGTAATAAATTCTTTATTTTTAATGAACACGCAA
TTGCTACTCCATTCCAAATTGGTGGGTTCTTAATGCATGGGTTCACAGTTTCCCTACGAT
ATGCTTACGGGTCCGTTTTGTTAAATACTGTTAACGTCTGCCTCCCATTTGTTAAGTGGA
CTAAATATTTACCAGGTGATGCTAAATTCAAGGAAAATGAAAAGACTCAATACAGTTTG
TTAGATTGGATTATTGAATGTATGCACCAGGCCAGTGCTCAAAGAGGTCAAAAGCTAA
GAGGCCCTCCATCTGCCAAGGATATCAATTTTTTCATTGACAAGAATAGAGACATTAAA
GATCTATTGAAGGGTTTGAAATGTTACAGACCATACATTAATTATTCTGTTAACCCAGAT
GGAACTCCAAAACCACCAAAAAAGATGCAAGCTAAGGGTATCGTTGGTTTCGTAAGA
GAGACTCCAGATTCTATGAAATTCAGAACTTTACCAAGTAACATGGAAAAGAATGGTG
TACCAAAACCAGGTGAAAAGGAAATCATGGTCACAACAACCGCCTACTTTGCCAAGA
AGTATGACATCAAATTGAAATACCCAGATGTTAAAATGGTGAGTCTAGGTGGTTCGAAT
GTTGTCCCAGCTGAATGTTTAACCATTGTTCCAGGTCAGAAATTAAAGGGTTTGGTTTA
TGACGAAAAAGCCGTTATTGATTTCACTGCTTTAAGACCAAGTGAAAAATTCAGAGCC
ATCACCAACTTGGCATTACCTGCCATTAAGAGAGCTTTATCAACTGAAGAAGAAAATGC
TAAGGCTCCACATGATTCTGGTTACACTTTCATGAAGGTCCCATCTCGTGTCATTGACG
CTCCTGTGGTTCAATTCAAGAACACTACAGTCACATATGTGGATAAACCATTTGGTACT

AAGAATGGTAAGAATAATCATGAAGAAACTAAGGGTAATTGGAATTTAAAGGATCATAA
ATTCATTACTGTTCCAAAGGAACCAATGCACTTGAGAGCTATCTTTATTAATGATTCTGA
TAAGTCTCCACCAGTTTCTGTCATGGATGAATTGAAGGCCTCTTTGAGCAAATTCGCTG
AAGATGTCGCTGATGTCGGTGTTAACTTTGACGTATCCATGGCCCCAATTTTAATTAACA
ATTTTAATGCCCCAATAAAGAAGGTTACTGGTGGCTTTGGTGGTAGAGGTGGACGTGG
TGGTAGAGGCGGCCGTGGTGGCCGTGGTGGTAGAGGAGGTCGTGGTGGTTTCGGCGG
TGGCCGTGGTGAAACTACTTATGAATTAACTCCAGGTGAAGAAAAACTACGTCACTTG
TTAGCCAACGTCCCAGAAAAGACTTATGTCTTATTCGTCTTGGGTCGTGGGGATGACTC
TGCTATTTACAACAGATTGAAATATTTAGCTGATTTGACGTACGGTGTCATTAACAATTG
TGTTATTTGGAACAAGTTCAGAAAGTGTTCCACTCAATACAATGTTAATGTGGTAATGA
AGATGAACTTGAAGTTAGAAGGTGCTAACCACTCCTTATGTGCAGAAGATATAAACTTA
CTGAAGGATGAAAAATCAGGATTACCATTCATGATTTTAGGTGCTGATGTTACACATTAT
CCAGAAAAGGATCAGAATTCTATCTCTGCATTGGTGGGTTCTTTTGATGACAAATTTGC
TCAATTCCCAGGTTCATATATGTTACAAAGTGGTCCAGGTGAAGAAATAATTGCTGGTAT
CGGTAACATGGTTCTACAGAGATTGAAACTGTATCAAAAGCACAACAATGGCAAACTA
CCTCCAAAGATTCTATTCTACAGAGATGGTGTTTCTGAATCCCAATTCTCACAAATTGTT
CAAATTGAAGTTAAGGGTTTGAAACAAGCATTGAAGAAATTCGGTAGTGAATTAAACA
AGGGTGTCAACTATAACCCATCTGTCACCACAATTTGTGTTGTCAAGAGAAATCAAATT
AGATTTATGCCACTTGAGCAAAATGCTATAAATGAAAAGGGTGAAGTTGCTGCCGTTCA
ATCATTCGAGAACGTTATGCCAGGTACTGTTGTTGATCGTGGTATCACCTCTAGTGCTCA
CTTCGATTTCTTCTTGCAATCTCAACAACCATTGAAGGGTACTGGTGTTCCATGTCATTA
CTGGTGTATCTACGACGAAAATCAATTCAATTCTGATTACTTGCAACAAGTTACTCACG
CTTTGTGTTACTTATTCGGTAGATCGAGTACAAGTATTAAGGTCGCATCTCCTGTTTATTA
CGCTGATTTATTGTGTGAACGTGGTGCTGCATTCTTCAAGGCTAACTTTGAGCTTGCTC
AATACGAATTTTCTAAGGAGAGGAAGAACAGAGATGACGTTATACCGACCGGTAAATT
ACTACAACCTGTTCATAAGAATGTCACTGACATCATGTACTACATATGA

pPICZA-EGFP-HIS4300-rad9-antiEGFP-HIS4300：

GATCTAACATCCAAAGACGAAAGGTTGAATGAAACCTTTTTGCCATCCGACATCCACA
GGTCCATTCTCACACATAAGTGCCAAACGCAACAGGAGGGGATACACTAGCAGCAGAC
CGTTGCAAACGCAGGACCTCCACTCCTCTTCTCCTCAACACCCACTTTTGCCATCGAA
AAACCAGCCCAGTTATTGGGCTTGATTGGAGCTCGCTCATTCCAATTCCTTCTATTAGGC
TACTAACACCATGACTTTATTAGCCTGTCTATCCTGGCCCCCCTGGCGAGGTTCATGTTT
GTTTATTTCCGAATGCAACAAGCTCCGCATTACACCCGAACATCACTCCAGATGAGGGC
TTTCTGAGTGTGGGGTCAAATAGTTTCATGTTCCCCAAATGGCCCAAAACTGACAGTTT
AAACGCTGTCTTGGAACCTAATATGACAAAAGCGTGATCTCATCCAAGATGAACTAAG
TTTGGTTCGTTGAAATGCTAACGGCCAGTTGGTCAAAAAGAAACTTCCAAAAGTCGGC
ATACCGTTTGTCTTGTTTGGTATTGATTGACGAATGCTCAAAAATAATCTCATTAATGCTT
AGCGCAGTCTCTCTATCGCTTCTGAACCCCGGTGCACCTGTGCCGAAACGCAAATGGG
GAAACACCCGCTTTTTGGATGATTATGCATTGTCTCCACATTGTATGCTTCCAAGATTCT
GGTGGGAATACTGCTGATAGCCTAACGTTCATGATCAAAATTTAACTGTTCTAACCCCT
ACTTGACAGCAATATATAAACAGAAGGAAGCTGCCCTGTCTTAAACCTTTTTTTTTATCA
TCATTATTAGCTTACTTTCATAATTGCGACTGGTTCCAATTGACAAGCTTTTGATTTTAAC
GACTTTTAACGACAACTTGAGAAGATCAAAAAACAACTAATTATTCGAAACGAGGAAT
TCATGGTGAGCAAGGGCGAGGAGCTGTTCACCGGGGTGGTGCCCATCCTGGTCGAGC
TGGACGGCGACGTAAACGGCCACAAGTTCAGCGTGTCCGGCGAGGGCGAGGGCGATG
CCACCTACGGCAAGCTGACCCTGAAGTTCATCTGCACCACCGGCAAGCTGCCCGTGCC
CTGGCCCACCCTCGTGACCACCCTGACCTACGGCGTGCAGTGCTTCAGCCGCTACCCC
GACCACATGAAGCAGCACGACTTCTTCAAGTCCGCCATGCCCGAAGGCTACGTCCAGG
AGCGCACCATCTTCTTCAATGACATTTCCCTTGCTACCTGCATACGCAAGTGTTGCAGA
GTTTGATAATTCCTTGAGTTTGGTAGGAAAAGCCGTGTTTCCCTATGCTGCTGACCAGC
TGCACAACCTGATCAAGTTCACTCAATCGACTGAGCTTCAAGTTAATGTGCAAGTTGA
GTCATCCGTTACAGAGGACCAATTTGAGGAGCTGATCGACAACTTGCTCAAGTTGTAC
AATAATGGTATCAATGAAGTGATTTTGGACCTAGATTTGGCAGAAAGAGTTGTCCAAAG
GATGATCCCAGGCGCTAGGGTTATAAAATCAGGTGTGTTGGAACTTTTTTCAAACCTTA
CTAAACATTGAAACTAATTGGTAAAGATAGAGGCGGCCGCATAACCCTAGCGCCTGGG
ATCATCCTTTGGACAACTCTTTCTGCCAAATCTAGGTCCAAAATCACTTCATTGATACCA
TTATTGTACAACTTGAGCAAGTTGTCGATCAGCTCCTCAAATTGGTCCTCTGTAACGGA
TGACTCAACTTGCACATTAACTTGAAGCTCAGTCGATTGAGTGAACTTGATCAGGTTGT
GCAGCTGGTCAGCAGCATAGGGAAACACGGCTTTTCCTACCAAACTCAAGGAATTATC
AAACTCTGCAACACTTGCGTATGCAGGTAGCAAGGGAAATGTCATTGAAGAAGATGGT
GCGCTCCTGGACGTAGCCTTCGGGCATGGCGGACTTGAAGAAGTCGTGCTGCTTCATG
TGGTCGGGGTAGCGGCTGAAGCACTGCACGCCGTAGGTCAGGGTGGTCACGAGGGTG
GGCCAGGGCACGGGCAGCTTGCCGGTGGTGCAGATGAACTTCAGGGTCAGCTTGCCG
TAGGTGGCATCGCCCTCGCCCTCGCCGGACACGCTGAACTTGTGGCCGTTTACGTCGC
CGTCCAGCTCGACCAGGATGGGCACCACCCCGGTGAACAGCTCCTCGCCCTTGCTCAC
CATACCGGTCTTGCTAGATTCTAATCAAGAGGATGTCAGAATGCCATTTGCCTGAGAGA
TGCAGGCTTCATTTTTGATACTTTTTTATTTGTAACCTATATAGTATAGGATTTTTTTTGTC
ATTTTGTTTCTTCTCGTACGAGCTTGCTCCTGATCAGCCTATCTCGCAGCTGATGAATAT
CTTGTGGTAGGGGTTTGGGAAAATCATTCGAGTTTGATGTTTTTCTTGGTATTTCCCACT
CCTCTTCAGAGTACAGAAGATTAAGTGAGACCTTCGTTTGTGCGGATCCCCCACACAC

CATAGCTTCAAAATGTTTCTACTCCTTTTTTACTCTTCCAGATTTTCTCGGACTCCGCGC
ATCGCCGTACCACTTCAAAACACCCAAGCACAGCATACTAAATTTCCCTCTTTCTTCC
TCTAGGGTGTCGTTAATTACCCGTACTAAAGGTTTGGAAAAGAAAAAAGAGACCGCCT
CGTTTCTTTTTCTTCGTCGAAAAAGGCAATAAAAATTTTTATCACGTTTCTTTTTCTTGA
AATTTTTTTTTTTAGTTTTTTTCTCTTTCAGTGACCTCCATTGATATTTAAGTTAATAAAC
GGTCTTCAATTTCTCAAGTTTCAGTTTCATTTTTCTTGTTCTATTACAACTTTTTTTACTT
CTTGTTCATTAGAAAGAAAGCATAGCAATCTAATCTAAGGGGCGGTGTTGACAATTAAT
CATCGGCATAGTATATCGGCATAGTATAATACGACAAGGTGAGGAACTAAACCATGGCC
AAGTTGACCAGTGCCGTTCCGGTGCTCACCGCGCGCGACGTCGCCGGAGCGGTCGAG
TTCTGGACCGACCGGCTCGGGTTCTCCCGGGACTTCGTGGAGGACGACTTCGCCGGTG
TGGTCCGGGACGACGTGACCCTGTTCATCAGCGCGGTCCAGGACCAGGTGGTGCCGG
ACAACACCCTGGCCTGGGTGTGGGTGCGCGGCCTGGACGAGCTGTACGCCGAGTGGT
CGGAGGTCGTGTCCACGAACTTCCGGGACGCCTCCGGGCCGGCCATGACCGAGATCG
GCGAGCAGCCGTGGGGGCGGGAGTTCGCCCTGCGCGACCCGGCCGGCAACTGCGTGC
ACTTCGTGGCCGAGGAGCAGGACTGACACGTCCGACGGCGGCCCACGGGTCCCAGGC
CTCGGAGATCCGTCCCCCTTTTCCTTTGTCGATATCATGTAATTAGTTATGTCACGCTTAC
ATTCACGCCCTCCCCCCACATCCGCTCTAACCGAAAAGGAAGGAGTTAGACAACCTGA
AGTCTAGGTCCCTATTTATTTTTTTATAGTTATGTTAGTATTAAGAACGTTATTTATATTTC
AAATTTTTCTTTTTTTTCTGTACAGACGCGTGTACGCATGTAACATTATACTGAAAACCT
TGCTTGAGAAGGTTTTGGGACGCTCGAAGGCTTTAATTTGCAAGCTGGAGACCAACAT
GTGAGCAAAAGGCCAGCAAAAGGCCAGGAACCGTAAAAAGGCCGCGTTGCTGGCGT
TTTTCCATAGGCTCCGCCCCCCTGACGAGCATCACAAAAATCGACGCTCAAGTCAGAG
GTGGCGAAACCCGACAGGACTATAAAGATACCAGGCGTTTCCCCCTGGAAGCTCCCTC
GTGCGCTCTCCTGTTCCGACCCTGCCGCTTACCGGATACCTGTCCGCCTTTCTCCCTTC
GGGAAGCGTGGCGCTTTCTCAATGCTCACGCTGTAGGTATCTCAGTTCGGTGTAGGTCG
TTCGCTCCAAGCTGGGCTGTGTGCACGAACCCCCCGTTCAGCCCGACCGCTGCGCCTT
ATCCGGTAACTATCGTCTTGAGTCCAACCCGGTAAGACACGACTTATCGCCACTGGCAG
CAGCCACTGGTAACAGGATTAGCAGAGCGAGGTATGTAGGCGGTGCTACAGAGTTCTT
GAAGTGGTGGCCTAACTACGGCTACACTAGAAGGACAGTATTTGGTATCTGCGCTCTGC
TGAAGCCAGTTACCTTCGGAAAAAGAGTTGGTAGCTCTTGATCCGGCAAACAAACCAC
CGCTGGTAGCGGTGGTTTTTTTGTTTGCAAGCAGCAGATTACGCGCAGAAAAAAAGGA
TCTCAAGAAGATCCTTTGATCTTTTCTACGGGGTCTGACGCTCAGTGGAACGAAAACT
CACGTTAAGGGATTTTGGTCATGAGATC

pPICZA-AOXTT-pAOX1-YPS1-pAOX1-AOXTT-His4：

CCTCGAGCGTTTCGAATAATTAGTTGTTTTTTGATCTTCTCAAGTTGTCGTTAAAAGTCG
TTAAAATCAAAAGCTTGTCAATTGGAACCAGTCGCAATTATGAAAGTAAGCTAATAATG
ATGATAAAAAAAAAAGGTTTAAGACAGGGCAGCTTCCTTCTGTTTATATATTGCTGTCAA
GTAGGGGTTAGAACAGTTAAATTTTGATCATGAACGTTAGGCTATCAGCAGTATTCCCA
CCAGAATCTTGGAAGCATACAATGTGGAGACAATGCATAATCATCCAAAAAGCGGGTG
TTTCCCCATTTGCGTTTCGGCACAGGTGCACCGGGGTTCAGAAGCGATAGAGAGACTG
CGCTAAGCATTAATGAGATTATTTTTGAGCATTCGTCAATCAATACCAAACAAGACAAA
CGGTATGCCGACTTTTGGAAGTTTCTTTTTGACCAACTGGCCGTTAGCATTTCAACGAA
CCAAACTTAGTTCATCTTGGATGAGATCACGCTTTTGTCATATTAGGTTCCAAGACAGC
GTTTAAACTGTCAGTTTTGGGCCATTTGGGGAACATGAAACTATTTGACCCCACACTCA
GAAAGCCCTCATCTGGAGTGATGTTCGGGTGTAATGCGGAGCTTGTTGCATTCGGAAAT
AAACAAACATGAACCTCGCCAGGGGGGCCAGGATAGACAGGCTAATAAAGTCATGGT
GTTAGTAGCCTAATAGAAGGAATTGGAATGAGCGAGCTCCAATCAAGCCCAATAACTG
GGCTGGTTTTTCGATGGCAAAAGTGGGTGTTGAGGAGAAGAGGAGTGGAGGTCCTGC
GTTTGCAACGGTCTGCTGCTAGTGTATCCCCTCCTGTTGCGTTTGGCACTTATGTGTGA
GAATGGACCTGTGGATGTCGGATGGCAAAAAGGTTTCATTCAACCTTTCGTCTTTGGAT
GTTAGATCTCAAGAGGATGTCAGAATGCCATTTGCCTGAGAGATGCAGGCTTCATTTTT
GATACTTTTTTATTTGTAACCTATATAGTATAGGATTTTTTTTGTCATTTTGTTTCTTCTCG
TACGAGCTTGCTCCTGATCAGCCTATCTCGCAGCTGATGAATATCTTGTGGTAGGGGTTT
GGGAAAATCATTCGAGTTTGATGTTTTTCTTGGTATTTCCCACTCCTCTTCAGAGTACAG
AAGATTAAGTGAGACCTTCGTTTGTGCAGCTGCCCCACACACCATAGCTTCAAAATGTT
TCTACTCCTTTTTTACTCTTCCAGATTTTCTCGGACTCCGCGCATCGCCGTACCACTTCA
AAACACCCAAGCACAGCATACTAAATTTCCCTCTTTCTTCCTCTAGGGTGTCGTTAATT
ACCCGTACTAAAGGTTTGGAAAAGAAAAAAGAGACCGCCTCGTTTCTTTTTCTTCGTC
GAAAAAGGCAATAAAAATTTTTATCACGTTTCTTTTTCTTGAAATTTTTTTTTTTAGTTTT
TTTCTCTTTCAGTGACCTCCATTGATATTTAAGTTAATAAACGGTCTTCAATTTCTCAAG
TTTCAGTTTCATTTTTCTTGTTCTATTACAACTTTTTTTACTTCTTGTTCATTAGAAAGAA
AGCATAGCAATCTAATCTAAGGGGCGGTGTTGACAATTAATCATCGGCATAGTATATCGG
CATAGTATAATACGACAAGGTGAGGAACTAAACCATGGCCAAGTTGACCAGTGCCGTT
CCGGTGCTCACCGCGCGCGACGTCGCCGGAGCGGTCGAGTTCTGGACCGACCGGCTC
GGGTTCTCCCGGGACTTCGTGGAGGACGACTTCGCCGGTGTGGTCCGGGACGACGTG
ACCCTGTTCATCAGCGCGGTCCAGGACCAGGTGGTGCCGGACAACACCCTGGCCTGG
GTGTGGGTGCGCGGCCTGGACGAGCTGTACGCCGAGTGGTCGGAGGTCGTGTCCACG
AACTTCCGGGACGCCTCCGGGCCGGCCATGACCGAGATCGGCGAGCAGCCGTGGGGG
CGGGAGTTCGCCCTGCGCGACCCGGCCGGCAACTGCGTGCACTTCGTGGCCGAGGAG
CAGGACTGACACGTCCGACGGCGGCCCACGGGTCCCAGGCCTCGGAGATCCGTCCCC
CTTTTCCTTTGTCGATATCATGTAATTAGTTATGTCACGCTTACATTCACGCCCTCCCCCC
ACATCCGCTCTAACCGAAAAGGAAGGAGTTAGACAACCTGAAGTCTAGGTCCCTATTT
ATTTTTTTATAGTTATGTTAGTATTAAGAACGTTATTTATATTTCAAATTTTTCTTTTTTTTC
TGTACAGACGCGTGTACGCATGTAACATTATACTGAAAACCTTGCTTGAGAAGGTTTTG
GGACGCTCGAAGGCTTTAATTTGCAAGCTGGAGACCAAGGCGGCCGCTTAAATAAGTC
CCAGTTTCTCCATACGAACCTTAACAGCATTGCGGTGAGCATCTAGACCTTCAACAGCA
GCCAGATCCATCACTGCTTGGCCAATATGTTTCAGTCCCTCAGGAGTTACGTCTTGTGA

AGTGATGAACTTCTGGAAGGTTGCAGTGTTAACTCCGCTGTATTGACGGGCATATCCGT
ACGTTGGCAAAGTGTGGTTGGTACCGGAGGAGTAATCTCCACAACTCTCTGGAGAGTA
GGCACCAACAAACACAGATCCAGCGTGTTGTACTTGATCAACATAAGAAGAAGCATTC
TCGATTTGCAGGATCAAGTGTTCAGGAGCGTACTGATTGGACATTTCCAAAGCCTGCTC
GTAGGTTGCAACCGATAGGGTTGTAGAGTGTGCAATACACTTGCGTACAATTTCAACCC
TTGGCAACTGCACAGCTTGGTTGTGAACAGCATCTTCAATTCTGGCAAGCTCCTTGTCT
GTCATATCGACAGCCAACAGAATCACCTGGGAATCAATACCATGTTCAGCTTGAGACA
GAAGGTCTGAGGCAACGAAATCTGGATCAGCGTATTTATCAGCAATAACTAGAACTTC
AGAAGGCCCAGCAGGCATGTCAATACTACACAGGGCTGATGTGTCATTTTGAACCATC
ATCTTGGCAGCAGTAACGAACTGGTTTCCTGGACCAAATATTTTGTCACACTTAGGAAC
AGTTTCTGTTCCGTAAGCCATAGCAGCTACTGCCTGGGCGCCTCCTGCTAGCACGATAC
ACTTAGCACCAACCTTGTGGGCAACGTAGATGACTTCTGGGGTAAGGGTACCATCCTT
CTTAGGTGGAGATGCAAAAACAATTTCTTTGCAACCAGCAACTTTGGCAGGAACACCC
AGCATCAGGGAAGTGGAAGGCAGAATTGCGGTTCCACCAGGAATATAGAGGCCAACT
TTCTCAATAGGTCTTGCAAAACGAGAGCAGACTACACCAGGGCAAGTCTCAACTTGCA
ACGTCTCCGTTAGTTGAGCTTCATGGAATTTCCTGACGTTATCTATAGAGAGATCAATGG
CTCTCTTAACGTTATCTGGCAATTGCATAAGTTCCTCTGGGAAAGGAGCTTCTAACACA
GGTGTCTTCAAAGCGACTCCATCAAACTTGGCAGTTAGTTCTAAAAGGGCTTTGTCAC
CATTTTGACGAACATTGTCGACAATTGGTTTGACTAATTCCATAATCTGTTCCGTTTTCT
GGATAGGACGACGAAGGGCATCTTCAATTTCTTGTGAGGAGGCCTTAGAAACGTCAAT
TTTGCACAATTCAATACGACCTTCAGAAGGGACTTCTTTAGGTTTGGATTCTTCTTTAG
GTTGTTCCTTGGTGTATCCTGGCTTGGCATCTCCTTTCCTTCTAGTGACCTTTAGGGACT
TCATATCCAGGTTTCTCTCCACCTCGTCCAACGTCACACCGTACTTGGCACATCTAACT
AATGCAAAATAAAATAAGTCAGCACATTCCCAGGCTATATCTTCCTTGGATTTAGCTTCT
GCAAGTTCATCAGCTTCCTCCCTAATTTTAGCGTTCAACAAAACTTCGTCGTCAAATAA
CCGTTTGGTATAAGAACCTTCTGGAGCATTGCTCTTACGATCCCACAAGGTGGCTTCCA
TGGCTCTAAGACCCTTTGATTGGCCAAAACAGGAAGTGCGTTCCAAGTGACAGAAAC
CAACACCTGTTTGTTCAACCACAAATTTCAAGCAGTCTCCATCACAATCCAATTCGATA
CCCAGCAACTTTTGAGTTGCTCCAGATGTAGCACCTTTATACCACAAACCGTGACGAC
GAGATTGGTAGACTCCAGTTTGTGTCCTTATAGCCTCCGGAATAGACTTTTTGGACGAG
TACACCAGGCCCAACGAGTAATTAGAAGAGTCAGCCACCAAAGTAGTGAATAGACCAT
CGGGGCGGTCAGTAGTCAAAGACGCCAACAAAATTTCACTGACAGGGAACTTTTTGA
CATCTTCAGAAAGTTCGTATTCAGTAGTCAATTGCCGAGCATCAATAATGGGGATTATAC
CAGAAGCAACAGTGGAAGTCACATCTACCAACTTTGCGGTCTCAGAAAAAGCATAAA
CAGTTCTACTACCGCCATTAGTGAAACTTTTCAAATCGCCCAGTGGAGAAGAAAAAGG
CACAGCGATACTAGCATTAGCGGGCAAGGATGCAACTTTATCAACCAGGGTCCTATAGA
TAACCCTAGCGCCTGGGATCATCCTTTGGACAACTCTTTCTGCCAAATCTAGGTCCAAA
ATCACTTCATTGATACCATTATTGTACAACTTGAGCAAGTTGTCGATCAGCTCCTCAAAT
TGGTCCTCTGTAACGGATGACTCAACTTGCACATTAACTTGAAGCTCAGTCGATTGAGT
GAACTTGATCAGGTTGTGCAGCTGGTCAGCAGCATAGGGAAACACGGCTTTTCCTACC
AAACTCAAGGAATTATCAAACTCTGCAACACTTGCGTATGCAGGTAGCAAGGGAAATG
TCATGAATTCATGTGAGCAAAAGGCCAGCAAAAGGCCAGGAACCGTAAAAAGGCCGC
GTTGCTGGCGTTTTTCCATAGGCTCCGCCCCCCTGACGAGCATCACAAAAATCGACGCT
CAAGTCAGAGGTGGCGAAACCCGACAGGACTATAAAGATACCAGGCGTTTCCCCCTGG

AAGCTCCCTCGTGCGCTCTCCTGTTCCGACCCTGCCGCTTACCGGATACCTGTCCGCCT
TTCTCCCTTCGGGAAGCGTGGCGCTTTCTCAATGCTCACGCTGTAGGTATCTCAGTTCG
GTGTAGGTCGTTCGCTCCAAGCTGGGCTGTGTGCACGAACCCCCCGTTCAGCCCGACC
GCTGCGCCTTATCCGGTAACTATCGTCTTGAGTCCAACCCGGTAAGACACGACTTATCG
CCACTGGCAGCAGCCACTGGTAACAGGATTAGCAGAGCGAGGTATGTAGGCGGTGCTA
CAGAGTTCTTGAAGTGGTGGCCTAACTACGGCTACACTAGAAGGACAGTATTTGGTATC
TGCGCTCTGCTGAAGCCAGTTACCTTCGGAAAAAGAGTTGGTAGCTCTTGATCCGGCA
AACAAACCACCGCTGGTAGCGGTGGTTTTTTTGTTTGCAAGCAGCAGATTACGCGCAG
AAAAAAAGGATCTCAAGAAGATCCTTTGATCTTTTCTACGGGGTCTGACGCTCAGTGG
AACGAAAACTCACGTTAAGGGATTTTGGTCATGAGATCAGCAGCTGCACAAACGAAG
GTCTCACTTAATCTTCTGTACTCTGAAGAGGAGTGGGAAATACCAAGAAAAACATCAA
ACTCGAATGATTTTCCCAAACCCCTACCACAAGATATTCATCAGCTGCGAGATAGGCTG
ATCAGGAGCAAGCTCGTACGAGAAGAAACAAAATGACAAAAAAAATCCTATACTATAT
AGGTTACAAATAAAAAAGTATCAAAAATGAAGCCTGCATCTCTCAGGCAAATGGCATT
CTGACATCCTCTTGAGATCTAACATCCAAAGACGAAAGGTTGAATGAAACCTTTTTGCC
ATCCGACATCCACAGGTCCATTCTCACACATAAGTGCCAAACGCAACAGGAGGGGATA
CACTAGCAGCAGACCGTTGCAAACGCAGGACCTCCACTCCTCTTCTCCTCAACACCCA
CTTTTGCCATCGAAAAACCAGCCCAGTTATTGGGCTTGATTGGAGCTCGCTCATTCCAA
TTCCTTCTATTAGGCTACTAACACCATGACTTTATTAGCCTGTCTATCCTGGCCCCCCTG
GCGAGGTTCATGTTTGTTTATTTCCGAATGCAACAAGCTCCGCATTACACCCGAACATC
ACTCCAGATGAGGGCTTTCTGAGTGTGGGGTCAAATAGTTTCATGTTCCCCAAATGGCC
CAAAACTGACAGTTTAAACGCTGTCTTGGAACCTAATATGACAAAAGCGTGATCTCATC
CAAGATGAACTAAGTTTGGTTCGTTGAAATGCTAACGGCCAGTTGGTCAAAAAGAAAC
TTCCAAAAGTCGGCATACCGTTTGTCTTGTTTGGTATTGATTGACGAATGCTCAAAAAT
AATCTCATTAATGCTTAGCGCAGTCTCTCTATCGCTTCTGAACCCCGGTGCACCTGTGCC
GAAACGCAAATGGGGAAACACCCGCTTTTTGGATGATTATGCATTGTCTCCACATTGTA
TGCTTCCAAGATTCTGGTGGGAATACTGCTGATAGCCTAACGTTCATGATCAAAATTTA
ACTGTTCTAACCCCTACTTGACAGCAATATATAAACAGAAGGAAGCTGCCCTGTCTTAA
ACCTTTTTTTTTATCATCATTATTAGCTTACTTTCATAATTGCGACTGGTTCCAATTGACA
AGCTTTTGATTTTAACGACTTTTAACGACAACTTGAGAAGATCAAAAAACAACTAATTA
TTCGAAACGCTCGAGGGATCACTCATCACTTCCAGAGCTGAATATTCCAATAAGACATT
GCGTGGCACTTCCCCCGGTGTAATATGTTGCAATCTGTAGATCGTACAGGGAAACTTCC
ACTGTAGCACCCCCAAAATTGAATACCAGAGAAGTGGTATCTGATGCAGAAACACAAC
GAATGGTATAAGCTTGTCTTGACGAATCATAGGAGCCGCCCAGGTTCCGGCCAATTGAA
TTTAAAACAGAAGAAGGAGCATACGTTAGCGTAGCTCCAGAGTCCAATAATGCAGCAA
ATCTCCCTTGTAAAAGAGTTCCCTGGTCAGAACCCTTTTTCACATCTATTCCATTTAAAG
TAATTTGTAAACGAATTGCCTCTCTGTAACCACTGGAAGCGAGTGTGTTGATGACTGGA
ACTGTCAACAATTGTCCCGAATATTTTTCATGATCCACACCTCCAAAGAGGATGGAACC
ACTTGAGGCGTCCTTGGAGTTCAAGTACAAGGAGTATGCATTTTTGTTGATCAACCCAT
CGGTGACCATCTTGGCTGGCAAATTGTCATACTGATACATTTCACTGACCGAAGATGCA
CTAGCATATGTGGATTCTAGGCCTTTCAGTCCAATACCTAACACACCAATAGAGGAATT
GGTCATGTCTGCAACGGCGAAGGAAAGTTCTTTCACCTCTATGCCGTCGATAATGACGT
CGTCATAACCCCAGATTCCTGAAGCAAAAGTGGTATCAGCGTATGAGATGAAAAAGTC
TGTATTATTGTCATGGAACGTCGAGGAATCAGCGTGATCAAACGTTCCATACGTAGAGC

AATCCAATGTTGCCTGTGCAGAACTTACCGATACGGCTGTGGCAGCACTACCACTACCA
CTACCACTACCACTACCACTACCACTACCAGTTGCCGTGGCGGTGGAAGGAGAACTAG
TGCTAGTTCCAACGAGCCAATCCCAAAAATTTTTATTCTGAGAAGTTTCATTTTTCTTGA
GATTGATGGGATCCCATTCGGCGATCTTCTCATCATGTATATCGGATC

pPICZA-AOXTT-pAOX1-YAP1-pAOX1-AOXTT-His4：

CCTCGAGCGTTTCGAATAATTAGTTGTTTTTTGATCTTCTCAAGTTGTCGTTAAAAGTCG
TTAAAATCAAAAGCTTGTCAATTGGAACCAGTCGCAATTATGAAAGTAAGCTAATAATG
ATGATAAAAAAAAAGGTTTAAGACAGGGCAGCTTCCTTCTGTTTATATATTGCTGTCAA
GTAGGGGTTAGAACAGTTAAATTTTGATCATGAACGTTAGGCTATCAGCAGTATTCCCA
CCAGAATCTTGGAAGCATACAATGTGGAGACAATGCATAATCATCCAAAAAGCGGGTG
TTTCCCCATTTGCGTTTCGGCACAGGTGCACCGGGGTTCAGAAGCGATAGAGAGACTG
CGCTAAGCATTAATGAGATTATTTTTGAGCATTCGTCAATCAATACCAAACAAGACAAA
CGGTATGCCGACTTTTGGAAGTTTCTTTTTGACCAACTGGCCGTTAGCATTTCAACGAA
CCAAACTTAGTTCATCTTGGATGAGATCACGCTTTTGTCATATTAGGTTCCAAGACAGC
GTTTAAACTGTCAGTTTTGGGCCATTTGGGGAACATGAAACTATTTGACCCCACACTCA
GAAAGCCCTCATCTGGAGTGATGTTCGGGTGTAATGCGGAGCTTGTTGCATTCGGAAAT
AAACAAACATGAACCTCGCCAGGGGGGCCAGGATAGACAGGCTAATAAAGTCATGGT
GTTAGTAGCCTAATAGAAGGAATTGGAATGAGCGAGCTCCAATCAAGCCCAATAACTG
GGCTGGTTTTTCGATGGCAAAAGTGGGTGTTGAGGAGAAGAGGAGTGGAGGTCCTGC
GTTTGCAACGGTCTGCTGCTAGTGTATCCCCTCCTGTTGCGTTTGGCACTTATGTGTGA
GAATGGACCTGTGGATGTCGGATGGCAAAAAGGTTTCATTCAACCTTTCGTCTTTGGAT
GTTAGATCTCAAGAGGATGTCAGAATGCCATTTGCCTGAGAGATGCAGGCTTCATTTTT
GATACTTTTTTATTTGTAACCTATATAGTATAGGATTTTTTTTGTCATTTTGTTTCTTCTCG
TACGAGCTTGCTCCTGATCAGCCTATCTCGCAGCTGATGAATATCTTGTGGTAGGGGTTT
GGGAAAATCATTCGAGTTTGATGTTTTTCTTGGTATTTCCCACTCCTCTTCAGAGTACAG
AAGATTAAGTGAGACCTTCGTTTGTGCAGCTGCCCCACACACCATAGCTTCAAAATGTT
TCTACTCCTTTTTTACTCTTCCAGATTTTCTCGGACTCCGCGCATCGCCGTACCACTTCA
AAACACCCAAGCACAGCATACTAAATTTCCCTCTTTCTTCCTCTAGGGTGTCGTTAATT
ACCCGTACTAAAGGTTTGGAAAAGAAAAAAGAGACCGCCTCGTTTCTTTTTCTTCGTC
GAAAAAGGCAATAAAAATTTTTATCACGTTTCTTTTTCTTGAAATTTTTTTTTTTAGTTTT
TTTCTCTTTCAGTGACCTCCATTGATATTTAAGTTAATAAACGGTCTTCAATTTCTCAAG
TTTCAGTTTCATTTTTCTTGTTCTATTACAACTTTTTTTACTTCTTGTTCATTAGAAAGAA
AGCATAGCAATCTAATCTAAGGGGCGGTGTTGACAATTAATCATCGGCATAGTATATCGG
CATAGTATAATACGACAAGGTGAGGAACTAAACCATGGCCAAGTTGACCAGTGCCGTT
CCGGTGCTCACCGCGCGCGACGTCGCCGGAGCGGTCGAGTTCTGGACCGACCGGCTC
GGGTTCTCCCGGGACTTCGTGGAGGACGACTTCGCCGGTGTGGTCCGGGACGACGTG
ACCCTGTTCATCAGCGCGGTCCAGGACCAGGTGGTGCCGGACAACACCCTGGCCTGG
GTGTGGGTGCGCGGCCTGGACGAGCTGTACGCCGAGTGGTCGGAGGTCGTGTCCACG
AACTTCCGGGACGCCTCCGGGCCGGCCATGACCGAGATCGGCGAGCAGCCGTGGGGG
CGGGAGTTCGCCCTGCGCGACCCGGCCGGCAACTGCGTGCACTTCGTGGCCGAGGAG
CAGGACTGACACGTCCGACGGCGGCCCACGGGTCCCAGGCCTCGGAGATCCGTCCCC
CTTTTCCTTTGTCGATATCATGTAATTAGTTATGTCACGCTTACATTCACGCCCTCCCCCC
ACATCCGCTCTAACCGAAAAGGAAGGAGTTAGACAACCTGAAGTCTAGGTCCCTATTT
ATTTTTTTATAGTTATGTTAGTATTAAGAACGTTATTTATATTTCAAATTTTTCTTTTTTTTC
TGTACAGACGCGTGTACGCATGTAACATTATACTGAAAACCTTGCTTGAGAAGGTTTTG
GGACGCTCGAAGGCTTTAATTTGCAAGCTGGAGACCAAGGCGGCCGCTTAAATAAGTC
CCAGTTTCTCCATACGAACCTTAACAGCATTGCGGTGAGCATCTAGACCTTCAACAGCA
GCCAGATCCATCACTGCTTGGCCAATATGTTTCAGTCCCTCAGGAGTTACGTCTTGTGA

AGTGATGAACTTCTGGAAGGTTGCAGTGTTAACTCCGCTGTATTGACGGGCATATCCGT
ACGTTGGCAAAGTGTGGTTGGTACCGGAGGAGTAATCTCCACAACTCTCTGGAGAGTA
GGCACCAACAAACACAGATCCAGCGTGTTGTACTTGATCAACATAAGAAGAAGCATTC
TCGATTTGCAGGATCAAGTGTTCAGGAGCGTACTGATTGGACATTTCCAAAGCCTGCTC
GTAGGTTGCAACCGATAGGGTTGTAGAGTGTGCAATACACTTGCGTACAATTTCAACCC
TTGGCAACTGCACAGCTTGGTTGTGAACAGCATCTTCAATTCTGGCAAGCTCCTTGTCT
GTCATATCGACAGCCAACAGAATCACCTGGGAATCAATACCATGTTCAGCTTGAGACA
GAAGGTCTGAGGCAACGAAATCTGGATCAGCGTATTTATCAGCAATAACTAGAACTTC
AGAAGGCCCAGCAGGCATGTCAATACTACACAGGGCTGATGTGTCATTTTGAACCATC
ATCTTGGCAGCAGTAACGAACTGGTTTCCTGGACCAAATATTTGTCACACTTAGGAAC
AGTTTCTGTTCCGTAAGCCATAGCAGCTACTGCCTGGGCGCCTCCTGCTAGCACGATAC
ACTTAGCACCAACCTTGTGGGCAACGTAGATGACTTCTGGGGTAAGGGTACCATCCTT
CTTAGGTGGAGATGCAAAAACAATTTCTTTGCAACCAGCAACTTTGGCAGGAACACCC
AGCATCAGGGAAGTGGAAGGCAGAATTGCGGTTCCACCAGGAATATAGAGGCCAACT
TTCTCAATAGGTCTTGCAAAACGAGAGCAGACTACACCAGGGCAAGTCTCAACTTGCA
ACGTCTCCGTTAGTTGAGCTTCATGGAATTTCCTGACGTTATCTATAGAGAGATCAATGG
CTCTCTTAACGTTATCTGGCAATTGCATAAGTTCCTCTGGGAAAGGAGCTTCTAACACA
GGTGTCTTCAAAGCGACTCCATCAAACTTGGCAGTTAGTTCTAAAAGGGCTTTGTCAC
CATTTTGACGAACATTGTCGACAATTGGTTTGACTAATTCCATAATCTGTTCCGTTTTCT
GGATAGGACGACGAAGGGCATCTTCAATTTCTTGTGAGGAGGCCTTAGAAACGTCAAT
TTTGCACAATTCAATACGACCTTCAGAAGGGACTTCTTTAGGTTTGGATTCTTCTTTAG
GTTGTTCCTTGGTGTATCCTGGCTTGGCATCTCCTTTCCTTCTAGTGACCTTTAGGGACT
TCATATCCAGGTTTCTCTCCACCTCGTCCAACGTCACACCGTACTTGGCACATCTAACT
AATGCAAAATAAAATAAGTCAGCACATTCCCAGGCTATATCTTCCTTGGATTTAGCTTCT
GCAAGTTCATCAGCTTCCTCCCTAATTTAGCGTTCAACAAAACTTCGTCGTCAAATAA
CCGTTTGGTATAAGAACCTTCTGGAGCATTGCTCTTACGATCCCACAAGGTGGCTTCCA
TGGCTCTAAGACCCTTTGATTGGCCAAAACAGGAAGTGCGTTCCAAGTGACAGAAAC
CAACACCTGTTTGTTCAACCACAAATTTCAAGCAGTCTCCATCACAATCCAATTCGATA
CCCAGCAACTTTTGAGTTGCTCCAGATGTAGCACCTTTATACCACAAACCGTGACGAC
GAGATTGGTAGACTCCAGTTTGTGTCCTTATAGCCTCCGGAATAGACTTTTTGGACGAG
TACACCAGGCCCAACGAGTAATTAGAAGAGTCAGCCACCAAAGTAGTGAATAGACCAT
CGGGGCGGTCAGTAGTCAAAGACGCCAACAAAATTTCACTGACAGGGAACTTTTTGA
CATCTTCAGAAAGTTCGTATTCAGTAGTCAATTGCCGAGCATCAATAATGGGGATTATAC
CAGAAGCAACAGTGGAAGTCACATCTACCAACTTTGCGGTCTCAGAAAAAGCATAAA
CAGTTCTACTACCGCCATTAGTGAAACTTTTCAAATCGCCCAGTGGAGAAGAAAAAGG
CACAGCGATACTAGCATTAGCGGGCAAGGATGCAACTTTATCAACCAGGGTCCTATAGA
TAACCCTAGCGCCTGGGATCATCCTTTGGACAACTCTTTCTGCCAAATCTAGGTCCAAA
ATCACTTCATTGATACCATTATTGTACAACTTGAGCAAGTTGTCGATCAGCTCCTCAAAT
TGGTCCTCTGTAACGGATGACTCAACTTGCACATTAACTTGAAGCTCAGTCGATTGAGT
GAACTTGATCAGGTTGTGCAGCTGGTCAGCAGCATAGGGAAACACGGCTTTTCCTACC
AAACTCAAGGAATTATCAAACTCTGCAACACTTGCGTATGCAGGTAGCAAGGGAAATG
TCATGAATTCATGTGAGCAAAAGGCCAGCAAAAGGCCAGGAACCGTAAAAAGGCCGC
GTTGCTGGCGTTTTTCCATAGGCTCCGCCCCCCTGACGAGCATCACAAAAATCGACGCT
CAAGTCAGAGGTGGCGAAACCCGACAGGACTATAAAGATACCAGGCGTTTCCCCCTGG

AAGCTCCCTCGTGCGCTCTCCTGTTCCGACCCTGCCGCTTACCGGATACCTGTCCGCCT
TTCTCCCTTCGGGAAGCGTGGCGCTTTCTCAATGCTCACGCTGTAGGTATCTCAGTTCG
GTGTAGGTCGTTCGCTCCAAGCTGGGCTGTGTGCACGAACCCCCCGTTCAGCCCGACC
GCTGCGCCTTATCCGGTAACTATCGTCTTGAGTCCAACCCGGTAAGACACGACTTATCG
CCACTGGCAGCAGCCACTGGTAACAGGATTAGCAGAGCGAGGTATGTAGGCGGTGCTA
CAGAGTTCTTGAAGTGGTGGCCTAACTACGGCTACACTAGAAGGACAGTATTTGGTATC
TGCGCTCTGCTGAAGCCAGTTACCTTCGGAAAAAGAGTTGGTAGCTCTTGATCCGGCA
AACAAACCACCGCTGGTAGCGGTGGTTTTTTTGTTTGCAAGCAGCAGATTACGCGCAG
AAAAAAAGGATCTCAAGAAGATCCTTTGATCTTTTCTACGGGGTCTGACGCTCAGTGG
AACGAAAACTCACGTTAAGGGATTTTGGTCATGAGATCAGCAGCTGCACAAACGAAG
GTCTCACTTAATCTTCTGTACTCTGAAGAGGAGTGGGAAATACCAAGAAAAACATCAA
ACTCGAATGATTTTCCCAAACCCCTACCACAAGATATTCATCAGCTGCGAGATAGGCTG
ATCAGGAGCAAGCTCGTACGAGAAGAAACAAAATGACAAAAAAAATCCTATACTATAT
AGGTTACAAATAAAAAAGTATCAAAAATGAAGCCTGCATCTCTCAGGCAAATGGCATT
CTGACATCCTCTTGAGATCTAACATCCAAAGACGAAAGGTTGAATGAAACCTTTTTGCC
ATCCGACATCCACAGGTCCATTCTCACACATAAGTGCCAAACGCAACAGGAGGGGATA
CACTAGCAGCAGACCGTTGCAAACGCAGGACCTCCACTCCTCTTCTCCTCAACACCCA
CTTTTGCCATCGAAAAACCAGCCCAGTTATTGGGCTTGATTGGAGCTCGCTCATTCCAA
TTCCTTCTATTAGGCTACTAACACCATGACTTTATTAGCCTGTCTATCCTGGCCCCCCTG
GCGAGGTTCATGTTTGTTTATTTCCGAATGCAACAAGCTCCGCATTACACCCGAACATC
ACTCCAGATGAGGGCTTTCTGAGTGTGGGGTCAAATAGTTTCATGTTCCCCAAATGGCC
CAAAACTGACAGTTTAAACGCTGTCTTGGAACCTAATATGACAAAAGCGTGATCTCATC
CAAGATGAACTAAGTTTGGTTCGTTGAAATGCTAACGGCCAGTTGGTCAAAAAGAAAC
TTCCAAAAGTCGGCATACCGTTTGTCTTGTTTGGTATTGATTGACGAATGCTCAAAAAT
AATCTCATTAATGCTTAGCGCAGTCTCTCTATCGCTTCTGAACCCCGGTGCACCTGTGCC
GAAACGCAAATGGGGAAACACCCGCTTTTTGGATGATTATGCATTGTCTCCACATTGTA
TGCTTCCAAGATTCTGGTGGGAATACTGCTGATAGCCTAACGTTCATGATCAAAATTTA
ACTGTTCTAACCCCTACTTGACAGCAATATATAAACAGAAGGAAGCTGCCCTGTCTTAA
ACCTTTTTTTTTATCATCATTATTAGCTTACTTTCATAATTGCGACTGGTTCCAATTGACA
AGCTTTTGATTTTAACGACTTTTAACGACAACTTGAGAAGATCAAAAAACAACTAATTA
TTCGAAACGCTCGAGGGATCCATGAGTGACGTGGTAAACAAGAGAGCGGCAACGTCC
AGCACGCAGACCAATAAGAGACAGGAACTCAGCTCGGCGCTGACAAAACCTGGAAG
GAAACCAGTGCAGACGGAACCCAAGGACAAAAGAACTGCCCAGAATAGAGCTGCTC
AACGGGCTTTCCGTGAGCGGAAAGAGAAGAAGATGAAAGAATTAGAAACCAAGGTTG
AAGAGCTAGAGAGACAAAAATCTCAGCTGAATACTGAAAGCGAATTTCTGCGATCCCA
GGTGGAAACATTAATTCATGAACTCTCAAAATATAGGGGAGAAACAGATGTACTAAGC
CTACTGCCGACAAGTATACCGCAGGAGAGTAAAAAAATGGTGAGAACTCCTAGTAGCA
ATACAACAAACTCCAGTTCTGTCGGGGTTACTCCGTCTTCTTCCACTCTGCGTTCGTCA
TCTAGTAGTGGAGTATATGAATTTCCCTGGAAGCTTTCTAATAGTCAAAATCCAAGTGG
AAGCAACAGCCCATTGGATCTCACTAAGGCAGGCCAACTACCGTCACCAACTTCTATC
AATCAGAATCCTGGGTTGACGGCCGAATCGGTGAAGAGCTCCTCGGATC

pPICZA-AOXTT-pAOX1-PRB1-pAOX1-AOXTT-His4：
TCGAGCGTTTCGAATAATTAGTTGTTTTTTGATCTTCTCAAGTTGTCGTTAAAAGTCGTT
AAAATCAAAAGCTTGTCAATTGGAACCAGTCGCAATTATGAAAGTAAGCTAATAATGAT
GATAAAAAAAAAGGTTTAAGACAGGGCAGCTTCCTTCTGTTTATATATTGCTGTCAAGT
AGGGGTTAGAACAGTTAAATTTTGATCATGAACGTTAGGCTATCAGCAGTATTCCCACC
AGAATCTTGGAAGCATACAATGTGGAGACAATGCATAATCATCCAAAAAGCGGGTGTT
TCCCCATTTGCGTTTCGGCACAGGTGCACCGGGGTTCAGAAGCGATAGAGAGACTGCG
CTAAGCATTAATGAGATTATTTTTGAGCATTCGTCAATCAATACCAAACAAGACAAACG
GTATGCCGACTTTTGGAAGTTTCTTTTTGACCAACTGGCCGTTAGCATTTCAACGAACC
AAACTTAGTTCATCTTGGATGAGATCACGCTTTTGTCATATTAGGTTCCAAGACAGCGT
TTAAACTGTCAGTTTTGGGCCATTTGGGGAACATGAAACTATTTGACCCCACACTCAGA
AAGCCCTCATCTGGAGTGATGTTCGGGTGTAATGCGGAGCTTGTTGCATTCGGAAATAA
ACAAACATGAACCTCGCCAGGGGGGCCAGGATAGACAGGCTAATAAAGTCATGGTGTT
AGTAGCCTAATAGAAGGAATTGGAATGAGCGAGCTCCAATCAAGCCCAATAACTGGGC
TGGTTTTTCGATGGCAAAAGTGGGTGTTGAGGAGAAGAGGAGTGGAGGTCCTGCGTT
TGCAACGGTCTGCTGCTAGTGTATCCCCTCCTGTTGCGTTTGGCACTTATGTGTGAGAA
TGGACCTGTGGATGTCGGATGGCAAAAAGGTTTCATTCAACCTTTCGTCTTTGGATGTT
AGATCTCAAGAGGATGTCAGAATGCCATTTGCCTGAGAGATGCAGGCTTCATTTTTGAT
ACTTTTTTATTTGTAACCTATATAGTATAGGATTTTTTTTGTCATTTTGTTTCTTCTCGTAC
GAGCTTGCTCCTGATCAGCCTATCTCGCAGCTGATGAATATCTTGTGGTAGGGGTTTGG
GAAAATCATTCGAGTTTGATGTTTTTCTTGGTATTTCCCACTCCTCTTCAGAGTACAGAA
GATTAAGTGAGACCTTCGTTTGTGCAGCTGCCCCACACACCATAGCTTCAAAATGTTTC
TACTCCTTTTTTACTCTTCCAGATTTTCTCGGACTCCGCGCATCGCCGTACCACTTCAAA
ACACCCAAGCACAGCATACTAAATTTTCCCTCTTTCTTCCTCTAGGGTGTCGTTAATTAC
CCGTACTAAAGGTTTGGAAAAGAAAAAAGAGACCGCCTCGTTTCTTTTTCTTCGTCGA
AAAAGGCAATAAAAATTTTTATCACGTTTCTTTTTCTTGAAATTTTTTTTTTTAGTTTTTT
TCTCTTTCAGTGACCTCCATTGATATTTAAGTTAATAAACGGTCTTCAATTTCTCAAGTT
TCAGTTTCATTTTTCTTGTTCTATTACAACTTTTTTTACTTCTTGTTCATTAGAAAGAAAG
CATAGCAATCTAATCTAAGGGGCGGTGTTGACAATTAATCATCGGCATAGTATATCGGCA
TAGTATAATACGACAAGGTGAGGAACTAAACCATGGCCAAGTTGACCAGTGCCGTTCC
GGTGCTCACCGCGCGCGACGTCGCCGGAGCGGTCGAGTTCTGGACCGACCGGCTCGG
GTTCTCCCGGGACTTCGTGGAGGACGACTTCGCCGGTGTGGTCCGGGACGACGTGAC
CCTGTTCATCAGCGCGGTCCAGGACCAGGTGGTGCCGGACAACACCCTGGCCTGGGT
GTGGGTGCGCGGCCTGGACGAGCTGTACGCCGAGTGGTCGGAGGTCGTGTCCACGAA
CTTCCGGGACGCCTCCGGGCCGGCCATGACCGAGATCGGCGAGCAGCCGTGGGGGCG
GGAGTTCGCCCTGCGCGACCCGGCCGGCAACTGCGTGCACTTCGTGGCCGAGGAGCA
GGACTGACACGTCCGACGGCGGCCCACGGGTCCCAGGCCTCGGAGATCCGTCCCCCT
TTTCCTTTGTCGATATCATGTAATTAGTTATGTCACGCTTACATTCACGCCCTCCCCCCAC
ATCCGCTCTAACCGAAAAGGAAGGAGTTAGACAACCTGAAGTCTAGGTCCCTATTTATT
TTTTTATAGTTATGTTAGTATTAAGAACGTTATTTATATTTCAAATTTTTCTTTTTTTTCTGT
ACAGACGCGTGTACGCATGTAACATTATACTGAAAACCTTGCTTGAGAAGGTTTTGGG
ACGCTCGAAGGCTTTAATTTGCAAGCTGGAGACCAAGGCGGCCGCTTAAATAAGTCCC
AGTTTCTCCATACGAACCTTAACAGCATTGCGGTGAGCATCTAGACCTTCAACAGCAGC
CAGATCCATCACTGCTTGGCCAATATGTTTCAGTCCCTCAGGAGTTACGTCTTGTGAAG

TGATGAACTTCTGGAAGGTTGCAGTGTTAACTCCGCTGTATTGACGGGCATATCCGTAC
GTTGGCAAAGTGTGGTTGGTACCGGAGGAGTAATCTCCACAACTCTCTGGAGAGTAGG
CACCAACAAACACAGATCCAGCGTGTTGTACTTGATCAACATAAGAAGAAGCATTCTC
GATTTGCAGGATCAAGTGTTCAGGAGCGTACTGATTGGACATTTCCAAAGCCTGCTCGT
AGGTTGCAACCGATAGGGTTGTAGAGTGTGCAATACACTTGCGTACAATTTCAACCCTT
GGCAACTGCACAGCTTGGTTGTGAACAGCATCTTCAATTCTGGCAAGCTCCTTGTCTG
TCATATCGACAGCCAACAGAATCACCTGGGAATCAATACCATGTTCAGCTTGAGACAG
AAGGTCTGAGGCAACGAAATCTGGATCAGCGTATTTATCAGCAATAACTAGAACTTCA
GAAGGCCCAGCAGGCATGTCAATACTACACAGGGCTGATGTGTCATTTTGAACCATCAT
CTTGGCAGCAGTAACGAACTGGTTTCCTGGACCAAATATTTTGTCACACTTAGGAACA
GTTTCTGTTCCGTAAGCCATAGCAGCTACTGCCTGGGCGCCTCCTGCTAGCACGATACA
CTTAGCACCAACCTTGTGGGCAACGTAGATGACTTCTGGGGTAAGGGTACCATCCTTCT
TAGGTGGAGATGCAAAAACAATTTCTTTGCAACCAGCAACTTTGGCAGGAACACCCAG
CATCAGGGAAGTGGAAGGCAGAATTGCGGTTCCACCAGGAATATAGAGGCCAACTTTC
TCAATAGGTCTTGCAAAACGAGAGCAGACTACACCAGGGCAAGTCTCAACTTGCAAC
GTCTCCGTTAGTTGAGCTTCATGGAATTTCCTGACGTTATCTATAGAGAGATCAATGGCT
CTCTTAACGTTATCTGGCAATTGCATAAGTTCCTCTGGGAAAGGAGCTTCTAACACAGG
TGTCTTCAAAGCGACTCCATCAAACTTGGCAGTTAGTTCTAAAAGGGCTTTGTCACCAT
TTTGACGAACATTGTCGACAATTGGTTTGACTAATTCCATAATCTGTTCCGTTTTCTGGA
TAGGACGACGAAGGGCATCTTCAATTTCTTGTGAGGAGGCCTTAGAAACGTCAATTTT
GCACAATTCAATACGACCTTCAGAAGGGACTTCTTTAGGTTTGGATTCTTCTTTAGGTT
GTTCCTTGGTGTATCCTGGCTTGGCATCTCCTTTCCTTCTAGTGACCTTTAGGGACTTCA
TATCCAGGTTTCTCTCCACCTCGTCCAACGTCACACCGTACTTGGCACATCTAACTAAT
GCAAAATAAAATAAGTCAGCACATTCCCAGGCTATATCTTCCTTGGATTTAGCTTCTGCA
AGTTCATCAGCTTCCTCCCTAATTTTAGCGTTCAACAAAACTTCGTCGTCAAATAACCG
TTTGGTATAAGAACCTTCTGGAGCATTGCTCTTACGATCCCACAAGGTGGCTTCCATGG
CTCTAAGACCCTTTGATTGGCCAAAACAGGAAGTGCGTTCCAAGTGACAGAAACCAA
CACCTGTTTGTTCAACCACAAATTTCAAGCAGTCTCCATCACAATCCAATTCGATACCC
AGCAACTTTTGAGTTGCTCCAGATGTAGCACCTTTATACCACAAACCGTGACGACGAG
ATTGGTAGACTCCAGTTTGTGTCCTTATAGCCTCCGGAATAGACTTTTTGGACGAGTAC
ACCAGGCCCAACGAGTAATTAGAAGAGTCAGCCACCAAAGTAGTGAATAGACCATCG
GGGCGGTCAGTAGTCAAAGACGCCAACAAAATTTCACTGACAGGGAACTTTTTGACAT
CTTCAGAAAGTTCGTATTCAGTAGTCAATTGCCGAGCATCAATAATGGGGATTATACCA
GAAGCAACAGTGGAAGTCACATCTACCAACTTTGCGGTCTCAGAAAAAGCATAAACA
GTTCTACTACCGCCATTAGTGAAACTTTTCAAATCGCCCAGTGGAGAAGAAAAAGGCA
CAGCGATACTAGCATTAGCGGGCAAGGATGCAACTTTATCAACCAGGGTCCTATAGATA
ACCCTAGCGCCTGGGATCATCCTTTGGACAACTCTTTCTGCCAAATCTAGGTCCAAAAT
CACTTCATTGATACCATTATTGTACAACTTGAGCAAGTTGTCGATCAGCTCCTCAAATTG
GTCCTCTGTAACGGATGACTCAACTTGCACATTAACTTGAAGCTCAGTCGATTGAGTGA
ACTTGATCAGGTTGTGCAGCTGGTCAGCAGCATAGGGAAACACGGCTTTTCCTACCAA
ACTCAAGGAATTATCAAACTCTGCAACACTTGCGTATGCAGGTAGCAAGGGAAATGTC
ATGAATTCATGTGAGCAAAAGGCCAGCAAAAGGCCAGGAACCGTAAAAAGGCCGCGT
TGCTGGCGTTTTTCCATAGGCTCCGCCCCCCTGACGAGCATCACAAAAATCGACGCTC
AAGTCAGAGGTGGCGAAACCCGACAGGACTATAAAGATACCAGGCGTTTCCCCCTGG

AAGCTCCCTCGTGCGCTCTCCTGTTCCGACCCTGCCGCTTACCGGATACCTGTCCGCCT
TTCTCCCTTCGGGAAGCGTGGCGCTTTCTCAATGCTCACGCTGTAGGTATCTCAGTTCG
GTGTAGGTCGTTCGCTCCAAGCTGGGCTGTGTGCACGAACCCCCCGTTCAGCCCGACC
GCTGCGCCTTATCCGGTAACTATCGTCTTGAGTCCAACCCGGTAAGACACGACTTATCG
CCACTGGCAGCAGCCACTGGTAACAGGATTAGCAGAGCGAGGTATGTAGGCGGTGCTA
CAGAGTTCTTGAAGTGGTGGCCTAACTACGGCTACACTAGAAGGACAGTATTTGGTATC
TGCGCTCTGCTGAAGCCAGTTACCTTCGGAAAAAGAGTTGGTAGCTCTTGATCCGGCA
AACAAACCACCGCTGGTAGCGGTGGTTTTTTTGTTTGCAAGCAGCAGATTACGCGCAG
AAAAAAAGGATCTCAAGAAGATCCTTTGATCTTTTCTACGGGGTCTGACGCTCAGTGG
AACGAAAACTCACGTTAAGGGATTTTGGTCATGAGATCAGCAGCTGCACAAACGAAG
GTCTCACTTAATCTTCTGTACTCTGAAGAGGAGTGGGAAATACCAAGAAAAACATCAA
ACTCGAATGATTTTCCCAAACCCCTACCACAAGATATTCATCAGCTGCGAGATAGGCTG
ATCAGGAGCAAGCTCGTACGAGAAGAAACAAAATGACAAAAAAAATCCTATACTATAT
AGGTTACAAATAAAAAAGTATCAAAAATGAAGCCTGCATCTCTCAGGCAAATGGCATT
CTGACATCCTCTTGAGATCTAACATCCAAAGACGAAAGGTTGAATGAAACCTTTTTGCC
ATCCGACATCCACAGGTCCATTCTCACACATAAGTGCCAAACGCAACAGGAGGGGATA
CACTAGCAGCAGACCGTTGCAAACGCAGGACCTCCACTCCTCTTCTCCTCAACACCCA
CTTTTGCCATCGAAAAACCAGCCCAGTTATTGGGCTTGATTGGAGCTCGCTCATTCCAA
TTCCTTCTATTAGGCTACTAACACCATGACTTTATTAGCCTGTCTATCCTGGCCCCCCTG
GCGAGGTTCATGTTTGTTTATTTCCGAATGCAACAAGCTCCGCATTACACCCGAACATC
ACTCCAGATGAGGGCTTTCTGAGTGTGGGGTCAAATAGTTTCATGTTCCCCAAATGGCC
CAAAACTGACAGTTTAAACGCTGTCTTGGAACCTAATATGACAAAAGCGTGATCTCATC
CAAGATGAACTAAGTTTGGTTCGTTGAAATGCTAACGGCCAGTTGGTCAAAAAGAAAC
TTCCAAAAGTCGGCATACCGTTTGTCTTGTTTGGTATTGATTGACGAATGCTCAAAAAT
AATCTCATTAATGCTTAGCGCAGTCTCTCTATCGCTTCTGAACCCCGGTGCACCTGTGCC
GAAACGCAAATGGGGAAACACCCGCTTTTTGGATGATTATGCATTGTCTCCACATTGTA
TGCTTCCAAGATTCTGGTGGGAATACTGCTGATAGCCTAACGTTCATGATCAAAATTTA
ACTGTTCTAACCCCTACTTGACAGCAATATATAAACAGAAGGAAGCTGCCCTGTCTTAA
ACCTTTTTTTTTATCATCATTATTAGCTTACTTTCATAATTGCGACTGGTTCCAATTGACA
AGCTTTTGATTTTAACGACTTTTAACGACAACTTGAGAAGATCAAAAAACAACTAATTA
TTCGAAACGCTCGAGTCGGTGGCAAATACTCTCGAATCCCTTTCAATGAAAGCAACTG
CTGGATGCTTACTGATAAGCTCTAAAGTGTCATCCGTGAAGTATCCGGTGTAACCCTTG
AGCAAGTCACCAACGGAGAAGTCACCGGTGTAACCTGTTTCGCCATTCTCATTAGAAA
CGAAAAATGAGTGATTTGGATCTGACTCTCTTAATTTGCTCACAGACTCTTCATGAAGA
GTGGAGATAAGGTCACGGTGGAAGTCGATATCCTCTGAAGTGACACCCTTCTTGAAGA
CTATAATATAAGAGTAAGGGATTAAGTTTTTAGAAGCGGAATGGGTAGTAATGACTGGA
GCCGAAGGGTGGCCTTCTTTCATCTTCTTCAAAGCTTCAGGGCAAAGCTTAGGGTGTC
CCTTGGAATCCTTAGCGATTTTAGGATGCTTTCCATGGTGAGCCATATCAACTTTAGGAT
GTTTACCATGGTGGGCTAATACACCTTGTTCACTGTCACCATTAGCACCAAACTGGCTG
GGTAATGACTCAATGTTAGGAATTAGCAATCCTTGGACTGCTATGGCAGATAAGATAGC
CAATCCAACGGAATGACGCAATTGCATC

pPICZA-AOXTT-pAOX1-PEP4-pAOX1-AOXTT-His4：

CCTCGAGCGTTTCGAATAATTAGTTGTTTTTTGATCTTCTCAAGTTGTCGTTAAAAGTCG
TTAAAATCAAAAGCTTGTCAATTGGAACCAGTCGCAATTATGAAAGTAAGCTAATAATG
ATGATAAAAAAAAAAGGTTTAAGACAGGGCAGCTTCCTTCTGTTTATATATTGCTGTCAA
GTAGGGGTTAGAACAGTTAAATTTTGATCATGAACGTTAGGCTATCAGCAGTATTCCCA
CCAGAATCTTGGAAGCATACAATGTGGAGACAATGCATAATCATCCAAAAAGCGGGTG
TTTCCCCATTTGCGTTTCGGCACAGGTGCACCGGGGTTCAGAAGCGATAGAGAGACTG
CGCTAAGCATTAATGAGATTATTTTTGAGCATTCGTCAATCAATACCAAACAAGACAAA
CGGTATGCCGACTTTTGGAAGTTTCTTTTTGACCAACTGGCCGTTAGCATTTCAACGAA
CCAAACTTAGTTCATCTTGGATGAGATCACGCTTTTGTCATATTAGGTTCCAAGACAGC
GTTTAAACTGTCAGTTTTGGGCCATTTGGGGAACATGAAACTATTTGACCCCACACTCA
GAAAGCCCTCATCTGGAGTGATGTTCGGGTGTAATGCGGAGCTTGTTGCATTCGGAAAT
AAACAAACATGAACCTCGCCAGGGGGGCCAGGATAGACAGGCTAATAAAGTCATGGT
GTTAGTAGCCTAATAGAAGGAATTGGAATGAGCGAGCTCCAATCAAGCCCAATAACTG
GGCTGGTTTTTCGATGGCAAAAGTGGGTGTTGAGGAGAAGAGGAGTGGAGGTCCTGC
GTTTGCAACGGTCTGCTGCTAGTGTATCCCCTCCTGTTGCGTTTGGCACTTATGTGTGA
GAATGGACCTGTGGATGTCGGATGGCAAAAAGGTTTCATTCAACCTTTCGTCTTTGGAT
GTTAGATCTCAAGAGGATGTCAGAATGCCATTTGCCTGAGAGATGCAGGCTTCATTTTT
GATACTTTTTTATTTGTAACCTATATAGTATAGGATTTTTTTTGTCATTTTGTTTCTTCTCG
TACGAGCTTGCTCCTGATCAGCCTATCTCGCAGCTGATGAATATCTTGTGGTAGGGGTTT
GGGAAAATCATTCGAGTTTGATGTTTTTCTTGGTATTTCCCACTCCTCTTCAGAGTACAG
AAGATTAAGTGAGACCTTCGTTTGTGCAGCTGCCCCACACACCATAGCTTCAAAATGTT
TCTACTCCTTTTTTACTCTTCCAGATTTTCTCGGACTCCGCGCATCGCCGTACCACTTCA
AAACACCCAAGCACAGCATACTAAATTTCCCTCTTTCTTCCTCTAGGGTGTCGTTAATT
ACCCGTACTAAAGGTTTGGAAAAGAAAAAAGAGACCGCCTCGTTTCTTTTTCTTCGTC
GAAAAAGGCAATAAAAATTTTTATCACGTTTCTTTTTCTTGAAATTTTTTTTTTTAGTTTT
TTTCTCTTTCAGTGACCTCCATTGATATTTAAGTTAATAAACGGTCTTCAATTTCTCAAG
TTTCAGTTTCATTTTTCTTGTTCTATTACAACTTTTTTTACTTCTTGTTCATTAGAAAGAA
AGCATAGCAATCTAATCTAAGGGGCGGTGTTGACAATTAATCATCGGCATAGTATATCGG
CATAGTATAATACGACAAGGTGAGGAACTAAACCATGGCCAAGTTGACCAGTGCCGTT
CCGGTGCTCACCGCGCGCGACGTCGCCGGAGCGGTCGAGTTCTGGACCGACCGGCTC
GGGTTCTCCCGGGACTTCGTGGAGGACGACTTCGCCGGTGTGGTCCGGGACGACGTG
ACCCTGTTCATCAGCGCGGTCCAGGACCAGGTGGTGCCGGACAACACCCTGGCCTGG
GTGTGGGTGCGCGGCCTGGACGAGCTGTACGCCGAGTGGTCGGAGGTCGTGTCCACG
AACTTCCGGGACGCCTCCGGGCCGGCCATGACCGAGATCGGCGAGCAGCCGTGGGGG
CGGGAGTTCGCCCTGCGCGACCCGGCCGGCAACTGCGTGCACTTCGTGGCCGAGGAG
CAGGACTGACACGTCCGACGGCGGCCCACGGGTCCCAGGCCTCGGAGATCCGTCCCC
CTTTTCCTTTGTCGATATCATGTAATTAGTTATGTCACGCTTACATTCACGCCCTCCCCCC
ACATCCGCTCTAACCGAAAAGGAAGGAGTTAGACAACCTGAAGTCTAGGTCCCTATTT
ATTTTTTTATAGTTATGTTAGTATTAAGAACGTTATTTATATTTCAAATTTTTCTTTTTTTTC
TGTACAGACGCGTGTACGCATGTAACATTATACTGAAAACCTTGCTTGAGAAGGTTTTG
GGACGCTCGAAGGCTTTAATTTGCAAGCTGGAGACCAAGGCGGCCGCTTAAATAAGTC
CCAGTTTCTCCATACGAACCTTAACAGCATTGCGGTGAGCATCTAGACCTTCAACAGCA
GCCAGATCCATCACTGCTTGGCCAATATGTTTCAGTCCCTCAGGAGTTACGTCTTGTGA

AGTGATGAACTTCTGGAAGGTTGCAGTGTTAACTCCGCTGTATTGACGGGCATATCCGT
ACGTTGGCAAAGTGTGGTTGGTACCGGAGGAGTAATCTCCACAACTCTCTGGAGAGTA
GGCACCAACAAACACAGATCCAGCGTGTTGTACTTGATCAACATAAGAAGAAGCATTC
TCGATTTGCAGGATCAAGTGTTCAGGAGCGTACTGATTGGACATTTCCAAAGCCTGCTC
GTAGGTTGCAACCGATAGGGTTGTAGAGTGTGCAATACACTTGCGTACAATTTCAACCC
TTGGCAACTGCACAGCTTGGTTGTGAACAGCATCTTCAATTCTGGCAAGCTCCTTGTCT
GTCATATCGACAGCCAACAGAATCACCTGGGAATCAATACCATGTTCAGCTTGAGACA
GAAGGTCTGAGGCAACGAAATCTGGATCAGCGTATTTATCAGCAATAACTAGAACTTC
AGAAGGCCCAGCAGGCATGTCAATACTACACAGGGCTGATGTGTCATTTTGAACCATC
ATCTTGGCAGCAGTAACGAACTGGTTTCCTGGACCAAATATTTTGTCACACTTAGGAAC
AGTTTCTGTTCCGTAAGCCATAGCAGCTACTGCCTGGGCGCCTCCTGCTAGCACGATAC
ACTTAGCACCAACCTTGTGGGCAACGTAGATGACTTCTGGGGTAAGGGTACCATCCTT
CTTAGGTGGAGATGCAAAAACAATTTCTTTGCAACCAGCAACTTTGGCAGGAACACCC
AGCATCAGGGAAGTGGAAGGCAGAATTGCGGTTCCACCAGGAATATAGAGGCCAACT
TTCTCAATAGGTCTTGCAAAACGAGAGCAGACTACACCAGGGCAAGTCTCAACTTGCA
ACGTCTCCGTTAGTTGAGCTTCATGGAATTTCCTGACGTTATCTATAGAGAGATCAATGG
CTCTCTTAACGTTATCTGGCAATTGCATAAGTTCCTCTGGGAAAGGAGCTTCTAACACA
GGTGTCTTCAAAGCGACTCCATCAAACTTGGCAGTTAGTTCTAAAAGGGCTTTGTCAC
CATTTTGACGAACATTGTCGACAATTGGTTTGACTAATTCCATAATCTGTTCCGTTTTCT
GGATAGGACGACGAAGGGCATCTTCAATTTCTTGTGAGGAGGCCTTAGAAACGTCAAT
TTTGCACAATTCAATACGACCTTCAGAAGGGACTTCTTTAGGTTTGGATTCTTCTTTAG
GTTGTTCCTTGGTGTATCCTGGCTTGGCATCTCCTTTCCTTCTAGTGACCTTTAGGGACT
TCATATCCAGGTTTCTCTCCACCTCGTCCAACGTCACACCGTACTTGGCACATCTAACT
AATGCAAAATAAAATAAGTCAGCACATTCCCAGGCTATATCTTCCTTGGATTTAGCTTCT
GCAAGTTCATCAGCTTCCTCCCTAATTTTAGCGTTCAACAAAACTTCGTCGTCAAATAA
CCGTTTGGTATAAGAACCTTCTGGAGCATTGCTCTTACGATCCCACAAGGTGGCTTCCA
TGGCTCTAAGACCCTTTGATTGGCCAAAACAGGAAGTGCGTTCCAAGTGACAGAAAC
CAACACCTGTTTGTTCAACCACAAATTTCAAGCAGTCTCCATCACAATCCAATTCGATA
CCCAGCAACTTTTGAGTTGCTCCAGATGTAGCACCTTTATACCACAAACCGTGACGAC
GAGATTGGTAGACTCCAGTTTGTGTCCTTATAGCCTCCGGAATAGACTTTTTGGACGAG
TACACCAGGCCCAACGAGTAATTAGAAGAGTCAGCCACCAAAGTAGTGAATAGACCAT
CGGGGCGGTCAGTAGTCAAAGACGCCAACAAAATTTCACTGACAGGGAACTTTTTGA
CATCTTCAGAAAGTTCGTATTCAGTAGTCAATTGCCGAGCATCAATAATGGGGATTATAC
CAGAAGCAACAGTGGAAGTCACATCTACCAACTTTGCGGTCTCAGAAAAAGCATAAA
CAGTTCTACTACCGCCATTAGTGAAACTTTTCAAATCGCCCAGTGGAGAAGAAAAAGG
CACAGCGATACTAGCATTAGCGGGCAAGGATGCAACTTTATCAACCAGGGTCCTATAGA
TAACCCTAGCGCCTGGGATCATCCTTTGGACAACTCTTTCTGCCAAATCTAGGTCCAAA
ATCACTTCATTGATACCATTATTGTACAACTTGAGCAAGTTGTCGATCAGCTCCTCAAAT
TGGTCCTCTGTAACGGATGACTCAACTTGCACATTAACTTGAAGCTCAGTCGATTGAGT
GAACTTGATCAGGTTGTGCAGCTGGTCAGCAGCATAGGGAAACACGGCTTTTCCTACC
AAACTCAAGGAATTATCAAACTCTGCAACACTTGCGTATGCAGGTAGCAAGGGAAATG
TCATGAATTCATGTGAGCAAAAGGCCAGCAAAAGGCCAGGAACCGTAAAAAGGCCGC
GTTGCTGGCGTTTTTCCATAGGCTCCGCCCCCCTGACGAGCATCACAAAAATCGACGCT
CAAGTCAGAGGTGGCGAAACCCGACAGGACTATAAAGATACCAGGCGTTTCCCCCTGG

AAGCTCCCTCGTGCGCTCTCCTGTTCCGACCCTGCCGCTTACCGGATACCTGTCCGCCT
TTCTCCCTTCGGGAAGCGTGGCGCTTTCTCAATGCTCACGCTGTAGGTATCTCAGTTCG
GTGTAGGTCGTTCGCTCCAAGCTGGGCTGTGTGCACGAACCCCCCGTTCAGCCCGACC
GCTGCGCCTTATCCGGTAACTATCGTCTTGAGTCCAACCCGGTAAGACACGACTTATCG
CCACTGGCAGCAGCCACTGGTAACAGGATTAGCAGAGCGAGGTATGTAGGCGGTGCTA
CAGAGTTCTTGAAGTGGTGGCCTAACTACGGCTACACTAGAAGGACAGTATTTGGTATC
TGCGCTCTGCTGAAGCCAGTTACCTTCGGAAAAAGAGTTGGTAGCTCTTGATCCGGCA
AACAAACCACCGCTGGTAGCGGTGGTTTTTTTGTTTGCAAGCAGCAGATTACGCGCAG
AAAAAAAGGATCTCAAGAAGATCCTTTGATCTTTTCTACGGGGTCTGACGCTCAGTGG
AACGAAAACTCACGTTAAGGGATTTTGGTCATGAGATCAGCAGCTGCACAAACGAAG
GTCTCACTTAATCTTCTGTACTCTGAAGAGGAGTGGGAAATACCAAGAAAAACATCAA
ACTCGAATGATTTTCCCAAACCCCTACCACAAGATATTCATCAGCTGCGAGATAGGCTG
ATCAGGAGCAAGCTCGTACGAGAAGAAACAAAATGACAAAAAAAATCCTATACTATAT
AGGTTACAAATAAAAAAGTATCAAAAATGAAGCCTGCATCTCTCAGGCAAATGGCATT
CTGACATCCTCTTGAGATCTAACATCCAAAGACGAAAGGTTGAATGAAACCTTTTTGCC
ATCCGACATCCACAGGTCCATTCTCACACATAAGTGCCAAACGCAACAGGAGGGGATA
CACTAGCAGCAGACCGTTGCAAACGCAGGACCTCCACTCCTCTTCTCCTCAACACCCA
CTTTTGCCATCGAAAAACCAGCCCAGTTATTGGGCTTGATTGGAGCTCGCTCATTCCAA
TTCCTTCTATTAGGCTACTAACACCATGACTTTATTAGCCTGTCTATCCTGGCCCCCCTG
GCGAGGTTCATGTTTGTTTATTTCCGAATGCAACAAGCTCCGCATTACACCCGAACATC
ACTCCAGATGAGGGCTTTCTGAGTGTGGGGTCAAATAGTTTCATGTTCCCCAAATGGCC
CAAAACTGACAGTTTAAACGCTGTCTTGGAACCTAATATGACAAAAGCGTGATCTCATC
CAAGATGAACTAAGTTTGGTTCGTTGAAATGCTAACGGCCAGTTGGTCAAAAAGAAAC
TTCCAAAAGTCGGCATACCGTTTGTCTTGTTTGGTATTGATTGACGAATGCTCAAAAAT
AATCTCATTAATGCTTAGCGCAGTCTCTCTATCGCTTCTGAACCCCGGTGCACCTGTGCC
GAAACGCAAATGGGGAAACACCCGCTTTTTGGATGATTATGCATTGTCTCCACATTGTA
TGCTTCCAAGATTCTGGTGGGAATACTGCTGATAGCCTAACGTTCATGATCAAAATTTA
ACTGTTCTAACCCCTACTTGACAGCAATATATAAACAGAAGGAAGCTGCCCTGTCTTAA
ACCTTTTTTTTTATCATCATTATTAGCTTACTTTCATAATTGCGACTGGTTCCAATTGACA
AGCTTTTGATTTTAACGACTTTTAACGACAACTTGAGAAGATCAAAAAACAACTAATTA
TTCGAAACGCTCGAGGGATCCCGTCAAATTTGCCAAAAGCGAAGGCCAACCCCGGCT
CCGATGTGGCCTCAGCAAAATCAACTTTGGGAATGGTCAAATCCCCAATTTGCAACAC
ATCCTGAGAAACATACCCTTCCATGGAACCGGATCCATACCTAATTTCAAAGCTACTAC
CATTCTTCTTATAAGTAGAAGACTCATCATGGTCATACTTAGCATGCAAGAAGCAAGCT
AATGATCCACAATCTTTGCTAGGAACCCATAAATTGGAGGATCCTGTGTCAAGAATCAC
CTTGAACGATTGTGGAGGGGTACCTAATGATACCTCAGTAAAATACTGAGCGTTAAGAT
AGTTTGTAAGTGGAGCATCATGCGAAGCTTCAACGGCAAAACCATCTTGCTGAGACAT
AAAATTCGACTTGGACAAAGCATTTTGTTCGTTGAACAGAGAAACATATTTATGTTCCA
GAGCAGAGACATACTGCCCAAAATTGGCCTCTTTTAAAGTTTCTGAGACTGGATGCTT
GTGTATCTTAGCAGAATGAACTTTGGCTTCAGCACCAATACCTAGAGTAGAGAGCAAA
CCAATGGCAATTGACATCGTAGTACCGTCAAATATCATGATC

pMD18T-PNSIV9-AOXTT-pAOX1-FAS1-pAOX1 -AOXTT：

GACGAAAGGGCCTCGTGATACGCCTATTTTTATAGGTTAATGTCATGATAATAATGGTTT
CTTAGACGTCAGGTGGCACTTTTCGGGGAAATGTGCGCGGAACCCCTATTTGTTTATTT
TTCTAAATACATTCAAATATGTATCCGCTCATGAGACAATAACCCTGATAAATGCTTCAAT
AATATTGAAAAAGGAAGAGTATGAGTATTCAACATTTCCGTGTCGCCCTTATTCCCTTTT
TTGCGGCATTTTGCCTTCCTGTTTTTGCTCACCCAGAAACGCTGGTGAAAGTAAAAGAT
GCTGAAGATCAGTTGGGTGCACGAGTGGGTTACATCGAACTGGATCTCAACAGCGGTA
AGATCCTTGAGAGTTTTCGCCCCGAAGAACGTTTTCCAATGATGAGCACTTTTAAAGTT
CTGCTATGTGGCGCGGTATTATCCCGTATTGACGCCGGGCAAGAGCAACTCGGTCGCCG
CATACACTATTCTCAGAATGACTTGGTTGAGTACTCACCAGTCACAGAAAAGCATCTTA
CGGATGGCATGACAGTAAGAGAATTATGCAGTGCTGCCATAACCATGAGTGATAACACT
GCGGCCAACTTACTTCTGACAACGATCGGAGGACCGAAGGAGCTAACCGCTTTTTTGC
ACAACATGGGGGATCATGTAACTCGCCTTGATCGTTGGGAACCGGAGCTGAATGAAGC
CATACCAAACGACGAGCGTGACACCACGATGCCTGTAGCAATGGCAACAACGTTGCGC
AAACTATTAACTGGCGAACTACTTACTCTAGCTTCCCGGCAACAATTAATAGACTGGAT
GGAGGCGGATAAAGTTGCAGGACCACTTCTGCGCTCGGCCCTTCCGGCTGGCTGGTTT
ATTGCTGATAAATCTGGAGCCGGTGAGCGTGGGTCTCGCGGTATCATTGCAGCACTGG
GGCCAGATGGTAAGCCCTCCCGTATCGTAGTTATCTACACGACGGGGAGTCAGGCAAC
TATGGATGAACGAAATAGACAGATCGCTGAGATAGGTGCCTCACTGATTAAGCATTGGT
AACTGTCAGACCAAGTTTACTCATATATACTTTAGATTGATTTAAAACTTCATTTTTAATT
TAAAAGGATCTAGGTGAAGATCCTTTTTGATAATCTCATGACCAAAATCCCTTAACGTG
AGTTTTCGTTCCACTGAGCGTCAGACCCCGTAGAAAAGATCAAAGGATCTTCTTGAGA
TCCTTTTTTTCTGCGCGTAATCTGCTGCTTGCAAACAAAAAAACCACCGCTACCAGCG
GTGGTTTGTTTGCCGGATCAAGAGCTACCAACTCTTTTTCCGAAGGTAACTGGCTTCAG
CAGAGCGCAGATACCAAATACTGTTCTTCTAGTGTAGCCGTAGTTAGGCCACCACTTCA
AGAACTCTGTAGCACCGCCTACATACCTCGCTCTGCTAATCCTGTTACCAGTGGCTGCT
GCCAGTGGCGATAAGTCGTGTCTTACCGGGTTGGACTCAAGACGATAGTTACCGGATA
AGGCGCAGCGGTCGGGCTGAACGGGGGGTTCGTGCACACAGCCCAGCTTGGAGCGA
ACGACCTACACCGAACTGAGATACCTACAGCGTGAGCTATGAGAAAGCGCCACGCTTC
CCGAAGGGAGAAAGGCGGACAGGTATCCGGTAAGCGGCAGGGTCGGAACAGGAGAG
CGCACGAGGGAGCTTCCAGGGGGAAACGCCTGGTATCTTTATAGTCCTGTCGGGTTTC
GCCACCTCTGACTTGAGCGTCGATTTTTGTGATGCTCGTCAGGGGGGCGGAGCCTATG
GAAAAACGCCAGCAACGCGGCCTTTTTACGGTTCCTGGCCTTTTGCTGGCCTTTTGCTC
ACATGTTCTTTCCTGCGTTATCCCCTGATTCTGTGGATAACCGTATTACCGCCTTTGAGT
GAGCTGATACCGCTCGCCGCAGCCGAACGACCGAGCGCAGCGAGTCAGTGAGCGAGG
AAGCGGAAGAGCGCCCAATACGCAAACCGCCTCTCCCCGCGCGTTGGCCGATTCATTA
ATGCAGCTGGCACGACAGGTTTCCCGACTGGAAAGCGGGCAGTGAGCGCAACGCAAT
TAATGTGAGTTAGCTCACTCATTAGGCACCCCAGGCTTTACACTTTATGCTTCCGGCTCG
TATGTTGTGTGGAATTGTGAGCGGATAACAATTTCACACAGGAAACAGCTATGACCATG
ATTACGAATTCGAGCTCTAAGAGCTCCACAATCGCCAAGCATTCGTTAAGCCATCCATC
TCAGTTAGGCCCACTTCGACCCGTCCACCCACCGGTTACTAGTAACTAACAGACACTAT
TCAATAGGCCAGCACAGAAATGAAATCTATCGCTTTCTGTTCCAAGTCACCTGTCAACC
TGTGATTATCCAGGGTGCTTGGTCGAATTGCACTTACAGCATAATTACGTTGTAAACGA
CATTTTCCTGGGAGATAACTACAAAAAAATCCCACTGGTATTGTAACCACCATGAAACT

GCCATTGAATACCAGATTAGGCAATCTCAACTTTCAAAGCGTTTTGGAGTCCAAGATTC
AGTTCAGGAGGAATTGATCACTCAGATGGTGTTGGTACCCTTGACGTTACCCTGTGATG
TCACAATGCTCAAGCCCCTGCTAAACTCTCTTTAGTGGGTGGTTTTGTAAATTTTATGGT
TCATTCAAGTGGAATTACTATGTGTAGGGCTTACGAACTCAGGTAAAGGGTTCCTTTATT
ACTTACATAGGTTATGTAGGTGTCAGAAAAAGGGGAGCAGACCGGACAGTACACTGTC
CTGCGGAAATCGAAACTGCCATACCTCCCCCTCCCCCTACTTATCCTATTTAGCCTTCCT
ACAATCGTCGGAAGGTTCTACAATTGAGCAGCTGCTATGCAGCGTCCAATATCCCCTAC
GTCCATGCTACAAAAAAAGTCCCCGAAGGAAACTTCAACGCCATCCTGAGCTTCAGAC
AAACGAAAACCCTCAGCTCAATAAAGAGAATCGACATTCTCGGGATTCATGGACACTT
GGGGCTGAATGATCCCTGAATTGTTCTCAACAAGTGGTCTTCAACCTTCCTTAGAATAT
CAGCTTGAATTTACGTTGACTTTCAAATTGCCCAACTTAACCTGGGTTTAGAAACACAA
TTCCTCATACAAGTTAAGAGAAGCTCAAAAGAGTTCTTCAATCAACGGAGAGAGTTCC
CGCTAGAACTGAAATTCCGGATCTCACTTAATCTTCTGTACTCTGAAGAGGAGTGGGA
AATACCAAGAAAAACATCAAACTCGAATGATTTTCCCAAACCCCTACCACAAGATATTC
ATCAGCTGCGAGATAGGCTGATCAGGAGCAAGCTCGTACGAGAAGAAACAAAATGAC
AAAAAAAATCCTATACTATATAGGTTACAAATAAAAAAGTATCAAAAATGAAGCCTGCA
TCTCTCAGGCAAATGGCATTCTGACATCCTCTTGAGATCTAACATCCAAAGACGAAAG
GTTGAATGAAACCTTTTTGCCATCCGACATCCACAGGTCCATTCTCACACATAAGTGCC
AAACGCAACAGGAGGGGATACACTAGCAGCAGACCGTTGCAAACGCAGGACCTCCAC
TCCTCTTCTCCTCAACACCCACTTTTGCCATCGAAAAACCAGCCCAGTTATTGGGCTTG
ATTGGAGCTCGCTCATTCCAATTCCTTCTATTAGGCTACTAACACCATGACTTTATTAGC
CTGTCTATCCTGGCCCCCCTGGCGAGGTTCATGTTTGTTTATTTCCGAATGCAACAAGC
TCCGCATTACACCCGAACATCACTCCAGATGAGGGCTTTCTGAGTGTGGGGTCAAATA
GTTTCATGTTCCCCAAATGGCCCAAAACTGACAGTTTAAACGCTGTCTTGGAACCTAAT
ATGACAAAAGCGTGATCTCATCCAAGATGAACTAAGTTTGGTTCGTTGAAATGCTAAC
GGCCAGTTGGTCAAAAAGAAACTTCCAAAAGTCGGCATACCGTTTGTCTTGTTTGGTA
TTGATTGACGAATGCTCAAAAATAATCTCATTAATGCTTAGCGCAGTCTCTCTATCGCTT
CTGAACCCCGGTGCACCTGTGCCGAAACGCAAATGGGGAAACACCCGCTTTTTGGATG
ATTATGCATTGTCTCCACATTGTATGCTTCCAAGATTCTGGTGGGAATACTGCTGATAGC
CTAACGTTCATGATCAAAATTTAACTGTTCTAACCCCTACTTGACAGCAATATATAAACA
GAAGGAAGCTGCCCTGTCTTAAACCTTTTTTTTTATCATCATTATTAGCTTACTTTCATAA
TTGCGACTGGTTCCAATTGACAAGCTTTTGATTTTAACGACTTTTAACGACAACTTGAG
AAGATCAAAAAACAACTAATTATTCGAAACGCTCGAGGGATCATTGAAAATCAAAGAA
AACTTAATCAAGAACTACTACCTGGGAAGAGCCCTTGTCTACCTGCCAGGTCAGCGTG
GCCTTGTATACCCTCCCTCGGCATTACTTAATGCTGGAAAGTCCGGCTCCGCTCAGATCT
ACGCCATATTTGGAGGTCAAGGTAACACCGATGACTATTTCGAAGAACTTAGAGATATT
TACCACATCTACCAAGGTCTCGTGTCAGACTTCGTTACCAAGGCCCAGTTGAAGTTAC
AAGAGCTCATCAGAACCACCCCTGAAACTGACAGAATATATACACAGGGTCTAGACCT
GATCAATTGGCTGGAAAACAAGGACAAGACTCCTGACCAACAACAATTGCTATCCATT
CCAATGTCATGTCCTCTAATTTGTGTTATCCAACTGTGTCATTACATTGTCACTTGCCGC
ATCTTAGGTATTACACCTGGTCAACTCAGAGATTCCTTGAAAGGTACTACAGGTCACTC
CCAAGGTCTGGTCACCGCTGTGGTCGTTTCTTCTGCTGATTCCTGGGAATCCTTTGAGA
AATTGGCGCTTCAGGCAGTTGAATTCATGTTCTACATTGGTGTTAGAGGACTACAGGAT
CCCTCGAGCGTTTCGAATAATTAGTTGTTTTTTGATCTTCTCAAGTTGTCGTTAAAAGTC

GTTAAAATCAAAAGCTTGTCAATTGGAACCAGTCGCAATTATGAAAGTAAGCTAATAAT
GATGATAAAAAAAAAGGTTTAAGACAGGGCAGCTTCCTTCTGTTTATATATTGCTGTCA
AGTAGGGGTTAGAACAGTTAAATTTTGATCATGAACGTTAGGCTATCAGCAGTATTCCC
ACCAGAATCTTGGAAGCATACAATGTGGAGACAATGCATAATCATCCAAAAAGCGGGT
GTTTCCCCATTTGCGTTTCGGCACAGGTGCACCGGGGTTCAGAAGCGATAGAGAGACT
GCGCTAAGCATTAATGAGATTATTTTTGAGCATTCGTCAATCAATACCAAACAAGACAA
ACGGTATGCCGACTTTTGGAAGTTTCTTTTTGACCAACTGGCCGTTAGCATTTCAACGA
ACCAAACTTAGTTCATCTTGGATGAGATCACGCTTTTGTCATATTAGGTTCCAAGACAG
CGTTTAAACTGTCAGTTTTGGGCCATTTGGGGAACATGAAACTATTTGACCCCACACTC
AGAAAGCCCTCATCTGGAGTGATGTTCGGGTGTAATGCGGAGCTTGTTGCATTCGGAA
ATAAACAAACATGAACCTCGCCAGGGGGGCCAGGATAGACAGGCTAATAAAGTCATGG
TGTTAGTAGCCTAATAGAAGGAATTGGAATGAGCGAGCTCCAATCAAGCCCAATAACT
GGGCTGGTTTTTCGATGGCAAAAGTGGGTGTTGAGGAGAAGAGGAGTGGAGGTCCTG
CGTTTGCAACGGTCTGCTGCTAGTGTATCCCCTCCTGTTGCGTTTGGCACTTATGTGTG
AGAATGGACCTGTGGATGTCGGATGGCAAAAAGGTTTCATTCAACCTTTCGTCTTTGGA
TGTTAGATCTCAAGAGGATGTCAGAATGCCATTTGCCTGAGAGATGCAGGCTTCATTTT
TGATACTTTTTTATTTGTAACCTATATAGTATAGGATTTTTTTTGTCATTTTGTTTCTTCTC
GTACGAGCTTGCTCCTGATCAGCCTATCTCGCAGCTGATGAATATCTTGTGGTAGGGGT
TTGGGAAAATCATTCGAGTTTGATGTTTTTCTTGGTATTTCCCACTCCTCTTCAGAGTAC
AGAAGATTAAGTGAGAGGCGCGCCCCCGGGGCTCAAACAGGTTCATTCCATGGACCCA
TTCAAACGCACTGAAAATCGGCACTACTTACATAAAACCCAAATATGATGTTGGAACAT
TTTGTAATACTGCACTTTATTCCTTCACCTTCCCACCATTATCTACTATCCAGTGCACCAT
CTGGATAGCATTGAGGCCGAAACACATGAAAGAAGTCAGAATGTTTTGGCAAGTTGTT
AAAACTATTCTAGTTTGAGCACACATACTCTGAGATCTGGTCGCCGTATTTTCGTCTGAT
AGTTGCGTCCAACTATCAAATGATTTAACACTGTGCTGCCCAGATCATGAAAGTAATCT
ACCGTGAACAGCTAGTAGCTTAATATAACCACTAACTAGGTAACATGCCCATTTCAAAG
ATAATCATATCATAGATATTTTTACGTTTGAAATGGGCATCACGAGCTGGAATTACTGAC
ATCTGCTGGAGATATAGTAGTACTGAAAGAACGGACAGAGATCGGACAGAAAGCAGA
AAGAAATCGGATGGCGGACCACAGTTGAGCAGGAAAGCACTGAACCCAACCCGGAA
AAGCTAAACGTGAATCTGATCAAAGATCAATTTTCATCCCATCTGCATTCGTCATGCAA
CTTCAGCTTAACGACTGAAGAGAAGCATCAGAGACCAAGGTTAGTCAGAAGACCTAC
CCCATGGACTGAAACTTCAAAGTCCAGGGTGAACTATCTTTGACTAAACTGAGCATCTT
AGTTAACAAAAAATCGGGCAGAGACCCAAGTTAGTCAGCAGACCTACCCCATGGACT
GAAACTTCAAAGTCCAGGGTGAACTATCTTCTACTAACCTGAGCATCTCAGTCAATAAT
ACAAACATCCGAGCAGGAGGTGTTCAATTGGTTCTCAAAATCACACTCTCTAGTCAGA
AGGCTAACATCGTGACTGTCATTCTAAGCGCATGCAACACTTAGCCAAGTCCCAAGATA
GTAACGTATCTACCACAATCCACTATAAGTTAGTAGGCGCCTGATGCGGTATTTTCTCCT
TACGCATCTGTGCGGTATTTCACACCGCATATGGTGCACTCTCAGTACAATCTGCTCTGA
TGCCGCATAGTTAAGCCAGCCCCGACACCCGCCAACACCCGCTGACGCGCCCTGACGG
GCTTGTCTGCTCCCGGCATCCGCTTACAGACAAGCTGTGACCGTCTCCGGGAGCTGCA
TGTGTCAGAGGTTTTCACCGTCATCACCGAAACGCGCGA
